# Community-directed mass drug administration is undermined by status seeking in friendship networks and inadequate trust in health advice networks

**DOI:** 10.1016/j.socscimed.2017.04.009

**Published:** 2017-06

**Authors:** Goylette F. Chami, Andreas A. Kontoleon, Erwin Bulte, Alan Fenwick, Narcis B. Kabatereine, Edridah M. Tukahebwa, David W. Dunne

**Affiliations:** a Department of Land Economy, University of Cambridge, Cambridge CB3 9EP, United Kingdom; b Department of Pathology, University of Cambridge, Cambridge CB2 1QP, United Kingdom; c Development Economics Group, Wageningen University, Wageningen 6706 KN, The Netherlands; d Schistosomiasis Control Initiative, Imperial College London, London, W2 1PG, United Kingdom; e Uganda Ministry of Health, Vector Control Division, Bilharzia and Worm Control Programme, Kampala, Uganda

**Keywords:** Uganda, Social networks, Mass drug administration, Coverage, Compliance

## Abstract

Over 1.9 billion individuals require preventive chemotherapy through mass drug administration (MDA). Community-directed MDA relies on volunteer community medicine distributors (CMDs) and their achievement of high coverage and compliance. Yet, it is unknown if village social networks influence effective MDA implementation by CMDs. In Mayuge District, Uganda, census-style surveys were conducted for 16,357 individuals from 3,491 households in 17 villages. Praziquantel, albendazole, and ivermectin were administered for one month in community-directed MDA to treat *Schistosoma mansoni*, hookworm, and lymphatic filariasis. Self-reported treatment outcomes, socioeconomic characteristics, friendship networks, and health advice networks were collected. We investigated systematically missed coverage and noncompliance. Coverage was defined as an eligible person being offered at least one drug by CMDs; compliance included ingesting at least one of the offered drugs. These outcomes were analyzed as a two-stage process using a Heckman selection model. To further assess if MDA through CMDs was working as intended, we examined the probability of accurate drug administration of 1) praziquantel, 2) both albendazole and ivermectin, and 3) all drugs. This analysis was conducted using bivariate Probit regression. Four indicators from each social network were examined: degree, betweenness centrality, closeness centrality, and the presence of a direct connection to CMDs. All models accounted for nested household and village standard errors. CMDs were more likely to offer medicines, and to accurately administer the drugs as trained by the national control programme, to individuals with high friendship degree (many connections) and high friendship closeness centrality (households that were only a short number of steps away from all other households in the network). Though high (88.59%), additional compliance was associated with directly trusting CMDs for health advice. Effective treatment provision requires addressing CMD biases towards influential, well-embedded individuals in friendship networks and utilizing health advice networks to increase village trust in CMDs.

## Highlights

• Evaluation of mass drug administration implementation using social networks.
• Evidence of social biases and status-seeking from community medicine distributors.
• Coverage and compliance with mass drug administration for 16,357 individuals.
• Impact of friendship and health advice networks on treatment probability.
• Overlap of network status and formal social status in rural villages.

## 1 Background

Over 1.9 billion people in 125 countries require mass drug administration (MDA) for at least one of seven neglected tropical diseases (NTDs) (Webster et al., 2014). NTDs persist in destitute areas with inadequate sanitation infrastructure and limited access to clean water. MDA is the distribution of donated preventive chemotherapies to whole target populations, i.e. both infected and uninfected individuals in areas predominantly at high risk of helminthic infections. MDA is the mainstay of treatment for these chronic communicable diseases (Molyneux et al., 2016) because of the low cost per treatment, success in controlling morbidity, lack of widely available vaccines, and limited access to formal health care in rural poor areas. Soil-transmitted helminths, lymphatic filariasis, and schistosomiasis are the most prevalent infections treated through MDA (World Health Organization, 2015). These infections caused, respectively, 1.76 million (hookworm), 2.08 million, and 2.61 million disability adjusted life years in 2015 (Global Burden of Disease Study, 2015 DALYs and HALE Collaborators, 2016).

In our study area of Mayuge District, Uganda, community-directed MDA is used to treat hookworm, lymphatic filariasis, and intestinal schistosomiasis (*Schistosoma mansoni*) (Chami et al., 2015, Chami et al., 2016). Each village selects two unpaid community medicine distributors (CMDs). These volunteers are responsible for moving door-to-door to deliver medicines to all eligible individuals aged 1 + years (World Health Organization, 2006). CMDs first administer praziquantel for *S. mansoni* in week one of MDA then revisit all households in week two of MDA to deliver both albendazole and ivermectin for hookworm and lymphatic filariasis. Community-directed MDA is the only method of delivering preventive chemotherapies to both adults and children. Consequently, CMDs play a pivotal role in large-scale treatment campaigns that aim to curb transmission and progress towards geographically eliminating infections (Fleming et al., 2016, Molyneux et al., 2016).

Community-directed implementation is the most widely used MDA approach. It is the cornerstone of the African Programme for Onchocerciasis Control (APOC) (Homeida et al., 2002) and is recommended as the principal strategy for lymphatic filariasis treatment (World Health Organization, 2000). Though, for schistosomiasis and soil-transmitted helminth control programmes including in Uganda, MDA is commonly implemented through primary schools where teachers treat children in attendance (World Health Organization, 2006). Due to over 50% *S. mansoni* prevalence in school-aged children, communitywide treatment for schistosomiasis has been ongoing since 2003 in our study area (Kabatereine et al., 2004).

Despite their central role in MDA, the behaviors of CMDs are poorly understood. CMDs are involved in several activities in addition to MDA, including vitamin distribution, febrile malaria management, and vaccination campaigns (Katabarwa et al., 2010b). These time constraints can limit the performance of CMDs (Fleming et al., 2016, Katabarwa et al., 2010b). CMDs have neglected individuals for treatment due to the lack of monetary incentives (Fleming et al., 2016), differences in kinship (Katabarwa et al., 2010a), or being hard to reach (Parker et al., 2012). However, these reasons do not explain why individuals who are not provided treatment from CMDs also are the most socioeconomically disadvantaged (Chami et al., 2016, Njomo et al., 2012). There is a need to assess if CMDs prefer to treat particular individuals and hence, have an inherent social bias that affects MDA implementation. To do so, the decision of the CMD to offer medicine (coverage) must be separated from the choice of the recipient to ingest drugs (compliance). This separation (Shuford et al., 2016) enables a needed examination of how CMD performance (coverage) is associated with compliance. To our knowledge, no studies have examined MDA as a two-stage process.

Importantly, to explore potential social biases of CMDs during MDA implementation, social networks analyses are needed to reveal how CMDs interact with their community. It is widely established that the structure of personal interactions in a community affects the diffusion and adoption of public interventions (Bond et al., 2012, Borgatti et al., 2009, Centola, 2010, Rogers, 2003). Centrality is a set of heuristics that captures how well connected and embedded a node is in a network (Freeman, 1979). In social networks, centrality is an indicator of power or influence over other nodes (Hanneman and Riddle, 2005). For example, high degree nodes, i.e. with many connections, have been shown to be effective targets as opinion leaders who facilitate the diffusion/adoption of medical innovations (Valente and Davis, 1999). Though, targeting centrality for effectively seeding health interventions is debated (Centola, 2010, Kim et al., 2015, Valente and Davis, 1999). Other common centrality indicators include betweenness and closeness. The former indicator measures how often a node lies on the shortest paths connecting other nodes in a network whereas, closeness quantifies the shortest path distance from one node of interest to all other nodes in the network. These path-related centrality measures have been shown to be more relevant than formal training of hospital doctors in the Netherlands for the access to information and the adoption of a new feedback survey (Jippes et al., 2010). There are few complete network studies on health in low-income countries, see review in (Perkins et al., 2015). Several of these analyses focus on how social networks affect the participation of target populations during medical treatment, e.g. contraceptive use (Gayen and Raeside, 2010), or partaking in preventative health behaviours, e.g. latrine ownership (Shakya et al., 2015). The trend of examining demand-side aspects of health extends to published studies of MDA. The NTD community has focused on participant take up of MDA and cost-effectiveness of implementation (Kremer and Miguel, 2007, Krentel et al., 2013, Lo et al., 2015, Miguel and Kremer, 2004). There is a need to study the supply-side of MDA, i.e. what determines if and how drugs are offered by CMDs. Accordingly, though adoption and seeding of health interventions have been studied, an open question remains. How can social networks be utilized as an evaluation tool to understand if health programmes in low-income countries are being implemented as intended by policymakers?

We demonstrate how social network analyses can be used to evaluate social biases of CMDs during the implementation of MDA (Chami et al., 2013). A census-style survey was conducted for 16,357 individuals in 3,491 households of 17 villages. Friendship and health advice networks, self-reported treatment outcomes, and socioeconomic data were collected. We answer the following question. Is the implementation of MDA through CMDs working as intended and achieving unbiased communitywide treatment?

## 2 Methods

### 2.1 Participant sampling

On November 1st 2013—one month after MDA commenced—17 villages were visited within 5 km of Lake Victoria in Mayuge District, Uganda. Every household was approached and 97.57% (3491/3578) of all households were surveyed. In total, 87 households were either unavailable during the survey or refused to participate. Household heads and/or wives were interviewed. Data was collected on everyone eligible for MDA in the surveyed households (16,357 people), i.e. aged 1 + years.

### 2.2 Social networks

In total, 34 complete village friendship and health networks were surveyed. Two households were connected within a village if any person within those homes named or was named in response to a network prompt. Simple undirected graphs were generated. Network prompts were established in preliminary community focus groups. Nominations captured dependencies between households and thus applied to everyone within a household. Furthermore, networks were defined at the household instead of individual level because drug receipt is highly correlated within a home and CMDs move from home-to-home to deliver medicines (Chami et al., 2016).

#### 2.2.1 Close friendship networks prompt

“Please tell me the clan name first then the second name of up to 10 people that are very close friends to you. You should feel comfortable to turn to this person to borrow tools for fishing or farming without paying. A close friend is also someone that you see frequently. Do not name anyone in your household. Provide the names in the order of who is your closest friend first. Only name people in your village.”

#### 2.2.2 Health trust/advice networks prompt

“Please tell me the clan name first then the second name of up to 10 people that you trust for advice about taking drugs or any health problems. These people do not have to be health workers. Provide the names in the order of whose opinion you value most and who you would go to first. Only name people in your village.”

### 2.3 Network indicators

To investigate whether CMDs exhibit social biases during the implementation of MDA, we examined if and how the offer of treatment from CMDs varied based on individual centrality, i.e. network power or status. Individuals occupying central positions in the network have more influence over other members of the community (Hanneman and Riddle, 2005). Three common indicators of centrality were examined: degree, betweenness, and closeness. Degree was measured as the sum of incoming and outgoing edges, ignoring reciprocated edges. The natural log of degree plus one was used to account for village size and to capture the impact of large changes in degree, as it is right-skewed (Albert and Barabási, 2002). In addition to the association of connectivity and local influence (Valente and Davis, 1999), we were interested in degree as a potential indicator that can be easily monitored by MDA district health officers. In India, degree has been shown to be recognizable by village members (Banerjee et al., 2014). However, for social biases to manifest in centrality, it neither needs to be purposely targeted by CMDs nor directly visible to the community. Structural individualism theory in analytical sociology reveals that social networks can affect individual actions precisely because the network features are not recognizable (Hedstrom, 2005). Accordingly, we examined normalized indicators of betweenness (Brandes, 2001) and closeness (Freeman, 1979). Betweenness measures brokerage in a network, i.e. control over information flow between different sub-groups. Closeness centrality measures network embeddedness, and the direct access to information from and influence over all other nodes in the network. As we are studying medicine delivery, closeness centrality also may capture the ease in which individuals can be reached if CMDs follow the shortest network paths when moving from home to home during MDA (Borgatti, 2005). Lastly, we included dummy variables representing a direct network connection with a CMD.

### 2.4 Treatment outcomes

To investigate any systematic biases of CMDs and whom the CMDs chose to approach, treatment outcomes were constructed to measure if individuals were completely excluded from MDA. Coverage was defined as the offer of at least one drug of praziquantel, albendazole, or ivermectin to an eligible individual by CMDs. Compliance was defined as the acceptance and swallowing of at least one of any medicines offered; hence, compliance was only observed for individuals offered at least one drug by CMDs. To assess if MDA through CMDs worked as intended by the national control programme, two binary indicators of the actual drugs delivered by CMDs were constructed: an indicator for praziquantel and another indicator for the delivery of both albendazole and ivermectin. Hence, the offer of only one of albendazole or ivermectin was classified as an incorrect pill administration. CMDs were instructed during their training by vector control officers from the Ugandan Ministry of Health to deliver albendazole and ivermectin together in the second week of MDA. Additionally, all CMDs were trained in directly observed treatment (DOT) (Shuford et al., 2016), though data was unavailable to confirm if DOT was enforced.

### 2.5 Socioeconomic variables

Socioeconomic factors included age in years, gender, social status, occupation, education, religion, affiliation with the village majority tribe (binary), home latrine ownership (binary), home ownership (binary), and home quality score. Social status (binary) indicated if an individual previously or currently held a position as a religious, tribe or clan leader or on the local village council/government. Religion was a dummy variable and positive if the household head of a home was Muslim; the base category was Christian. Two dummy indicators for occupation were constructed: fishermen/fishmonger and other income-earning occupation. Fishermen and fishmongers were highlighted as it is debated whether these individuals are less likely to be offered treatment by CMDs during MDA (Chami et al., 2016, Parker et al., 2012). The base category for occupations included unemployed (non-income earning) adults, children, housewives, and students. Education was the highest level of education completed and was defined as follows: none (level 0); primary (levels 1–7); senior 1–6 (levels 8–13), diploma (level 14), some university (level 15), and completed university (level 16). Home quality score was the sum of the rank of floor, wall, and roof materials. Material rank order (1–4) was grass, sticks, plastic, and metal for the roof; mud/cow dung and sticks, plastic, metal, and bricks or cement for the walls; and mud/cow dung, plastic, wood planks, and brick or cement for the floor. Zero was recorded for a missing material.

### 2.6 Statistical analysis

The data were analyzed in Stata v13.1 (Stata Corp) and Python v2.7. Locally weighted regression (LOWESS smoothing) (Cleveland, 1979) was used to determine the functional form of the network variables against coverage (Supplementary Fig. 1). No clear relationship was observable between health closeness centrality and coverage, so this variable was not further investigated. Quadratic forms of friendship betweenness, friendship closeness, and health degree were used. These indicators were grand-mean centered to reduce collinearity and to aid in result interpretation. Marginal linear splines (Gould, 1993) were constructed for friendship degree and health betweenness centrality at the values of 3.526 and 0.268, respectively. The interval after the knot represents a change in slope of the predictor. To determine the inclusion of network indicators in the full models, univariate analyses against coverage (Supplementary Table 1) and checks of collinearity (Supplementary Table 2) were conducted (Valente et al., 2008). Health degree was insignificant in the univariate Probit regression (p-value > 0.05), so was not included in the full model. Friendship degree and friendship closeness were highly correlated (Spearman ρ 0.814, p-value < 0.05; Supplementary Table 2) and therefore analyzed in separate models whilst controlling for friendship and health betweenness centralities.

To examine systematically missed coverage and individual noncompliance, we modeled MDA as a two-stage process. An individual must first be offered medicine by CMDs before making a decision to adopt or reject what is offered. A nonrandom sample of individuals is offered medicines (Chami et al., 2016), thus adoption is only observed for a biased sample of the full network. We account for sample selection biases by studying coverage and compliance in a Heckman selection model (Heckman, 1979) with full maximum likelihood probit estimation in both stages. This regression framework accounts for unobserved correlation between errors in the selection (first stage) and outcome (second stage) equations. The dependent variable of the selection and outcome equations was coverage and compliance, respectively.

The main predictors for compliance were dummy indicators for having a direct connection to CMDs in either the health or friendship networks. In addition to these binary indicators, friendship degree, friendship closeness centrality, friendship betweenness centrality, and health betweenness centrality were predictors for coverage. Socioeconomic covariates were controlled for in both stages of the regression. Two exclusion criteria—variables that affect coverage (selection equation)—were included: the distance of the village centre to Lake Victoria and the total homes in each village. These criteria were selected because lakeside communities have a greater number of households than villages that do not border Lake Victoria (Chami et al., 2015). And, the number of households in a village has been shown to influence coverage (Fleming et al., 2016). Additional time is required by CMDs for MDA because, in our study area, there are only two CMDs per village irrespective of village size. Including these two village exclusion criteria significantly improved our model (Likelihood ratio tests: Degree-model Chi^2^ (2) = 409.16, Closeness-model Chi^2^ (2) = 405.51; p-values < 0.0001).

For the analysis of treatment offer for specific drugs, a bivariate probit model was used to simultaneously estimate two binary dependent variables and to account for correlation between the error terms (Cameron and Trivedi, 2010). The dependent variables were 1) praziquantel offer and 2) both albendazole and ivermectin administration. These outcomes represented the instructions provided to CMDs during training by the national control programme concerning how and in what order pills should be administered. Network indicators and covariates were specified as described for the Heckman selection model. As we were interested in MDA implementation with CMDs, we focused on the specific drugs offered for coverage as opposed to the decision of an individual to refuse a particular treatment.

In all regressions, we account for interdependencies between individuals and between households. Individuals (14,431) were nested in 3,387 households and 17 villages. These individuals included people who were eligible for at least one treatment and did not have missing information for any of the main predictors and covariates (Supplementary Tables 3-4). Standard errors were nested and clustered at both the household and village levels, with households as the primary sampling unit and villages as the strata (Judkins, 1990, Williams, 2000). A small sample correction was performed for the few clusters at the village level, as described in (Bester et al., 2011). We used clustered standard errors instead of random intercept models, since selection may manifest differently at each level, the balance of hierarchical data changes due to selection, and errors may not be equally correlated by level.

## 3 Results

### 3.1 Correlation of village coverage and compliance

**Figure 1:**
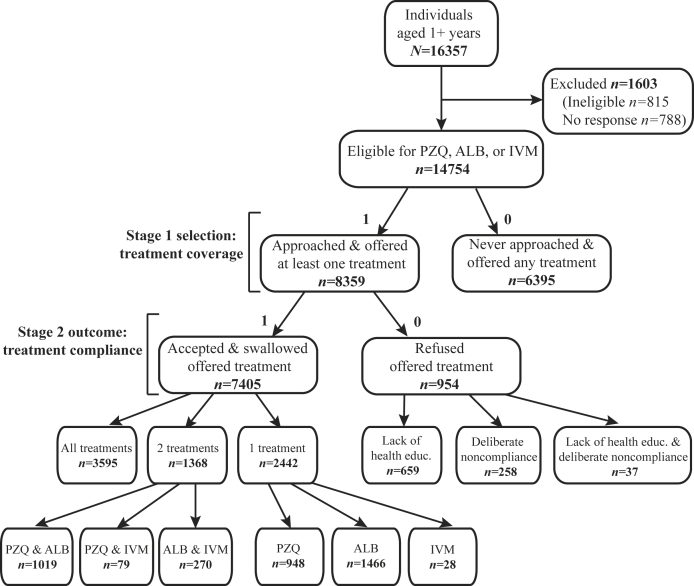
**Treatment outcomes for individuals in 17 study villages**. The distribution of praziquantel (PZQ), albendazole (ALB), and ivermectin (IVM) is provided for individuals aged one year and older. During mass drug administration, community medicine distributors informed 815 participants of ineligibility due to severe illness, pregnancy status (first trimester for IVM), or age (under five years for PZQ). No response was available for individuals where treatment offer was unknown. Stage 1 and 2 represent the sequence of actions for drug distribution. Individuals provided one or more reasons for noncompliance. For the lack of health education, individuals stated that they did not know the benefit and purpose of the drugs (71.32%, 470/659), did not need to take the drug because they had no symptoms (25.95%, 171/659), believed the drugs did not work (2.12%, 14/659), and witchcraft caused infections (1.67%, 11/659). Only 27.04% (258/954) of noncompliers deliberately refused treatment, despite knowledge of the drug benefits. Their responses noted bad side effects (66.28%, 171/258), lack of food or drink to accompany treatment (27.52%, 71/258), repurposing treatment for livestock (3.49%, 9/258), few friends and neighbors accepting treatment (3.49%, 9/258), and clan and tribe differences with the drug distributor (1.50%, 4/258). Only 3.88% (37/954) of individuals did not comply with treatment because of both the lack of health education and reasons of deliberate refusal.

**Figure 2:**
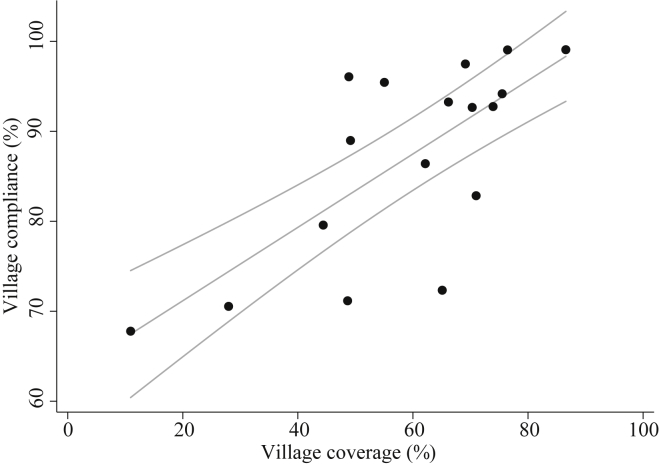
**Association of coverage and compliance across 17 villages**. The proportion of eligible individuals offered treatment (coverage) within each village was positively correlated to the proportion of those individuals who ultimately accepted and swallowed treatment (compliance). The fit of an ordinary least squares regression (Coeff. 0.408, p-value = <0.001; 95% CI 0.293, 0.523) is overlaid with the raw data points. The range of village coverage and compliance was 10.9%–86.6% and 67.8%–99.1%, respectively.

Fig. 1 presents a census of treatment outcomes for the population in the 17 study villages; household level outcomes are provided in Supplementary Figure 2. During one month of MDA, only 56.66% (8359/14754) of eligible individuals were approached and offered at least one treatment. Amongst the people approached, 88.59% (7405/8359) accepted and swallowed the offered treatment. The majority of noncompliance (69.08%, 659/954) was due to inadequate health education, including misinformation, about the distributed drugs. As shown in Fig. 2, village-level treatment coverage (% of all eligible people) and compliance (% of eligible people offered drugs) were positively and strongly correlated (Spearman ρ 0.691, p-value = 0.002*).* Socioeconomic characteristics of participants by treatment outcomes are provided in Supplementary Tables 3-5.

### 3.2 Network determinants of coverage

Table 1, Table 2 present the determinants of treatment from the Heckman selection model. The number of friendship edges was positively associated with the probability of being offered at least one drug by CMDs (p-value < 0.001). Additional friendship connections beyond 33 edges (97^th^ percentile) did not significantly increase the probability of coverage when compared to the probability of coverage for individuals with friendship degree of 33 (p-value > 0.05). An individual needed to have high friendship degree in order to have a higher likelihood of being offered treatment; it was not sufficient simply to be connected to other individuals with high friendship degree. Average neighbor friendship degree was uncorrelated with coverage (Obs. 14431, Spearman ρ 0.008, p-value = 0.340). The distance (geodesic paths) from one MDA recipient to all other individuals in the network was associated with the likelihood of treatment offer from CMDs. Friendship closeness centrality was positively correlated with coverage (p-value < 0.001). Concerning direct network connections, CMDs were 7–7.2% more likely to deliver drugs to individuals who trusted CMDs for health advice (p-value < 0.001). Though, being close friends with CMDs did not affect the likelihood of being approached and offered medicines by CMDs (p-value > 0.05).

**Table 1:** Average marginal effects (AME) of coverage and compliance for friendship degree Heckman model.

	*A) First stage dependent variable: coverage*					*B) Second stage dependent variable: compliance*				
Predictors	AME	Clustered Std. Err.	p-value	95% CI		AME | Coverage=1^a^	Clustered Std. Err.	p-value	95% CI	
LN(Friendship degree +1), [0, 3.526]	0.067	0.016	<0.001	0.036	0.099					
LN(Friendship degree +1), [>3.526]	0.303	0.232	0.193	−0.153	0.758					
Friendship betweenness centrality^b^	−1.513	1.073	0.158	−3.616	0.590					
Health betweenness centrality [0, 0.268]	0.420	0.350	0.231	−0.267	1.107					
Health betweenness centrality [>0.268]	0.520	0.769	0.499	−0.988	2.028					
Close friends with CMD	−0.004	0.020	0.846	−0.042	0.035	0.021	0.017	0.224	−0.013	0.054
Trusts CMD for health advice	0.072	0.016	<0.001	0.040	0.104	0.048	0.015	0.001	0.019	0.077
Age in years	0.001	<0.001	0.003	<0.001	0.002	<0.001	<0.001	0.546	<-0.001	0.001
Female	0.011	0.008	0.179	−0.005	0.027	0.011	0.008	0.143	−0.004	0.026
Social status: was or is a religious, tribe, or clan leader or on the local village council	0.074	0.026	0.005	0.022	0.125	0.018	0.025	0.476	−0.031	0.066
Fisherman or fishmonger	−0.018	0.019	0.338	−0.055	0.019	−0.002	0.016	0.907	−0.034	0.030
Other income-earning occupation	0.019	0.013	0.159	−0.007	0.045	0.027	0.013	0.029	0.003	0.052
Highest level of education attained	−0.009	0.002	<0.001	−0.012	−0.006	−0.006	0.002	<0.001	−0.009	−0.003
Muslim household head	−0.049	0.017	0.005	−0.082	−0.015	−0.021	0.015	0.149	−0.050	0.008
Household head belongs to village majority tribe	0.029	0.016	0.069	−0.002	0.060	0.038	0.014	0.008	0.010	0.066
No home latrine	−0.081	0.028	0.004	−0.136	−0.026	0.001	0.024	0.974	−0.047	0.049
Home owned, not rented	0.008	0.024	0.737	−0.039	0.055	0.002	0.023	0.934	−0.043	0.047
Home quality score	0.001	0.002	0.559	−0.003	0.006	−0.001	0.002	0.709	−0.005	0.004
Village centre greater than 0.50 km from Lake Victoria	−0.160	0.017	<0.001	−0.193	−0.127					
Total homes in village	−0.001	<0.001	<0.001	−0.001	−0.001					
Obs. 14431										
Households 3387										
Inverse hyperbolic tangent of ρ	−1.348	0.241	<0.001	−1.821	−0.875					
ρ	−0.874	0.057		−0.949	−0.704					
Wald test of independent equations, Chi^2^	31.100	<0.001								

a Average marginal probability of compliance = 1 conditional on coverage (being offered treatment).

b Entered in the Heckman selection model as grand-mean centered and a quadratic, i.e. also including the squared form.

**Table 2:** Average marginal effects (AME) of coverage and compliance for friendship closeness Heckman model.

	*A) First stage dependent variable: coverage*					*B) Second stage dependent variable: compliance*				
Predictors	AME	Clustered Std. Err.	p-value	95% CI		AME | Coverage=1^a^	Clustered Std. Err.	p-value	95% CI	
Friendship closeness centrality^b^	1.098	0.165	<0.001	0.775	1.421					
Friendship betweenness centrality^b^	−1.328	0.957	0.165	−3.205	0.548					
Health betweenness centrality [0, 0.268]	0.286	0.345	0.407	−0.390	0.963					
Health betweenness centrality [>0.268]	0.676	0.768	0.379	−0.831	2.182					
Close friends with CMD	−0.028	0.020	0.161	−0.068	0.011	0.016	0.017	0.362	−0.018	0.049
Trusts CMD for health advice	0.070	0.016	<0.001	0.038	0.102	0.048	0.015	0.001	0.019	0.077
Age in years	0.001	<0.001	0.003	<0.001	0.002	<0.001	<0.001	0.540	<-0.001	0.001
Female	0.010	0.008	0.197	−0.005	0.026	0.011	0.008	0.148	−0.004	0.026
Social status: was or is a religious, tribe, or clan leader or on the local village council	0.070	0.026	0.007	0.019	0.122	0.018	0.025	0.481	−0.031	0.067
Fisherman or fishmonger	−0.015	0.019	0.436	−0.051	0.022	−0.001	0.016	0.929	−0.033	0.030
Other income-earning occupation	0.017	0.013	0.203	−0.009	0.043	0.027	0.013	0.030	0.003	0.052
Highest level of education attained	−0.009	0.002	<0.001	−0.012	−0.006	−0.006	0.002	<0.001	−0.009	−0.003
Muslim household head	−0.043	0.017	0.011	−0.077	−0.010	−0.020	0.015	0.171	−0.049	0.009
Household head belongs to village majority tribe	0.029	0.016	0.064	−0.002	0.060	0.039	0.014	0.007	0.011	0.067
No home latrine	−0.079	0.028	0.005	−0.133	−0.024	0.001	0.025	0.961	−0.047	0.049
Home owned, not rented	0.013	0.024	0.579	−0.033	0.060	0.003	0.023	0.897	−0.042	0.048
Home quality score	0.002	0.002	0.444	−0.003	0.006	−0.001	0.002	0.723	−0.005	0.004
Village centre greater than 0.50 km from Lake Victoria	−0.170	0.017	<0.001	−0.203	−0.138					
Total homes in village	−0.001	<0.001	<0.001	−0.001	<-0.001					
Obs. 14431										
Households 3387										
Inverse hyperbolic tangent of ρ	−1.406	0.353	<0.001	−2.098	−0.714					
ρ	−0.887	0.076		−0.970	−0.613					
Wald test of independent equations, Chi^2^	15.84		<0.001							

a Average marginal probability of compliance = 1 conditional on coverage (being offered treatment).

b Entered in the Heckman selection model as grand-mean centered and a quadratic, i.e. also including the squared form.

**Figure 3:**
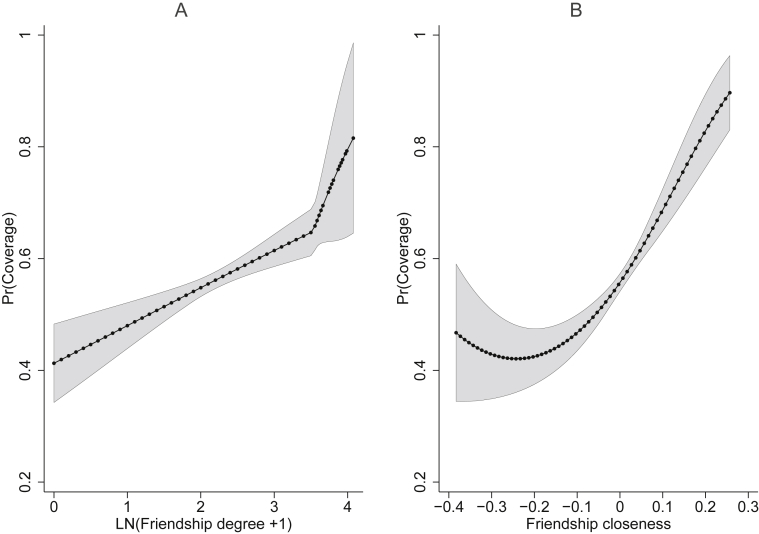
**Predicted probabilities of coverage at values of friendship degree and friendship closeness**. Predicted probabilities of coverage (the offer of at least one drug) against A) friendship degree and B) friendship closeness centrality are shown from the results of the Heckman selection models of Table 1, Table 2.

Fig. 3 shows the predicted probabilities of treatment offer at the range of values of friendship degree and friendship closeness. The individuals most likely to receive a treatment offer were in the most well-connected and well-embedded households of friendship networks. The probability of coverage was 0.658 for friendship degree ≥ 33 and a 0.897 for closeness centrality that was 0.257 greater than the mean.

When directed network indicators, which revealed who named whom, were used to predict coverage, all results were upheld (Supplementary Tables 6-7). Reexamining friendship degree in two parts—incoming versus outgoing edges—revealed that the likelihood of being offered medicines from CMDs was positively related to both the popularity (in-degree) and activity (out-degree) of the recipient's household. Concerning who trusted whom for health advice, individuals who named CMDs were more likely to be offered drugs during MDA when compared to individuals who did not name CMDs. In contrast, the individuals named by CMDs, i.e. people the CMDs go to for health advice, were not more likely to be offered medicines during MDA.

### 3.3 Accuracy of drug delivery by CMDs

**Figure 4:**
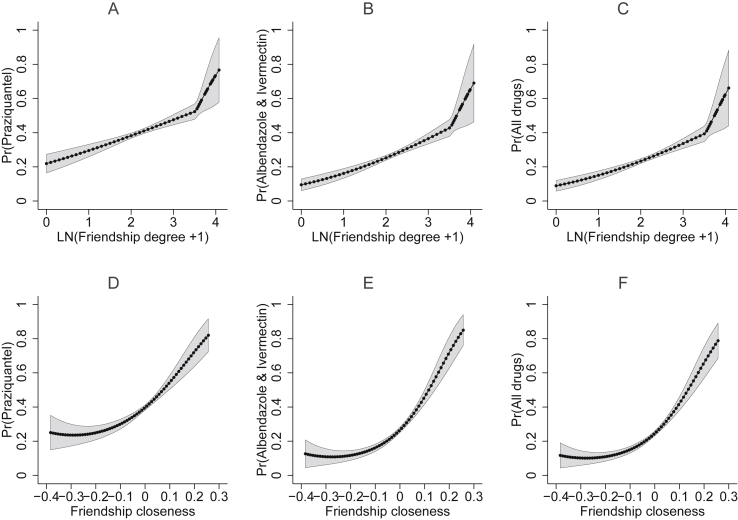
**Predicted probabilities of the accuracy of medicine delivery by CMDs at values of friendship degree and friendship closeness**. Panels A–C show the predicted probabilities from the bivariate probit model that includes friendship degree (Supplementary Table 8). Panels D–F present the predicted probabilities from the bivariate probit model with friendship closeness (Supplementary Table 9). Panels C and F from each model show the probabilities for both binary outcomes, 1) praziquantel and 2) both albendazole and ivermectin, equaling one.

Fig. 4 presents the effects of friendship degree and closeness on the probability of being offered particular combinations of pills from CMDs (Supplementary Tables 8-9). The probability of being offered praziquantel, both albendazole and ivermectin, or all three MDA drugs was positively associated with friendship degree and closeness. Remarkably, the accuracy of medicine administration also positively varied with friendship degree and closeness amongst only the individuals who were offered at least one drug (Supplementary Tables 10-11).

### 3.4 Overlap of friendship centrality and social status

In focus groups (Supplementary Data), CMDs indicated they became drug distributors to gain preferential health treatment at government health centers and social status within their village. Individuals with formal social status, i.e. village government members and religious, tribe or clan leaders, had a 0.07 higher probability (p-value < 0.05) of positive coverage than individuals with no such social status (Table 1, Table 2). However, unlike friendship degree and closeness, formal social status was not associated (p-value > 0.05) with being administered the correct combination of pills by CMDs (Supplementary Tables 8-11). There was an overlap of network status (high friendship degree or high friendship closeness) and formal social status (Supplementary Table 12). Individuals with social status had higher friendship degree and closeness when compared to individuals with no such status. This contrast was more pronounced when friendship degree and closeness were compared by household social status, which was defined as at least one eligible person of social status in the home. Interestingly, friendship degree, like formal social status, was a personal attribute that was recognizable by CMDs and the wider community (Supplementary Data).

### 3.5 Trust in CMDs and compliance

**Figure 5:**
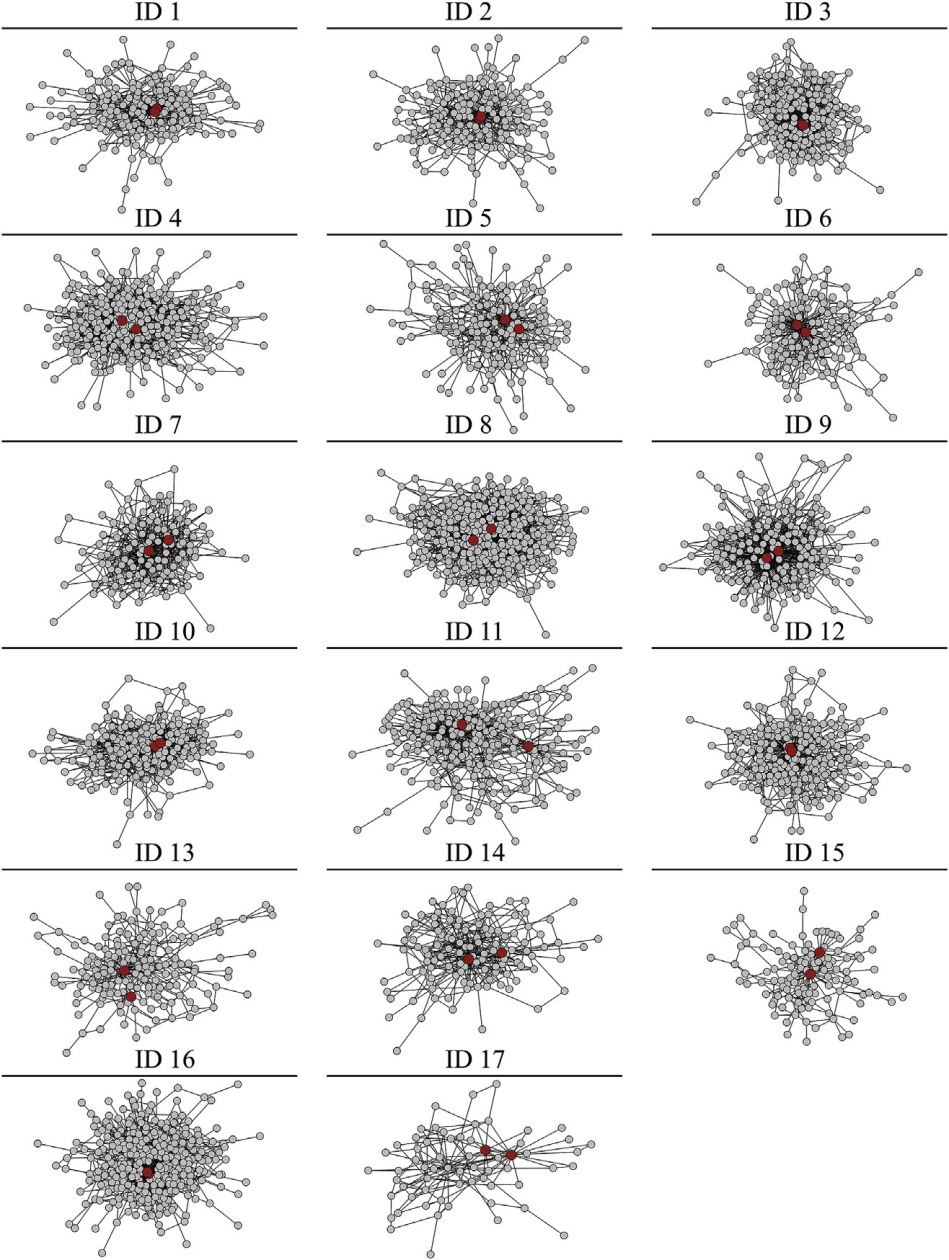
**Community medicine distributors and health advice networks**. Nodes represent households and edges represent trust for health advice. The largest component of each network is presented. Each ID represents one village. Two community medicine distributors (CMDs) are shown in red. The networks were drawn with a force-directed layout, so the most connected and embedded nodes were placed in the centre of the graph. IDs 1–3, 12, and 16 show two CMDs with similar network location.

CMDs were amongst the most trusted individuals for information about drugs and other health problems (Fig. 5), though CMDs were not necessarily the most connected individuals in their village health advice network. Overall, 38.96% (5748/14754) of all individuals eligible for MDA belonged to a household that named the CMD as someone trusted for health advice. After CMDs, the types of people most trusted for health advice, in order, included geographical neighbors, close friends, and village government members (Supplementary Table 13).

For anyone offered treatment, the average estimated probability of complying with treatment was high (0.878, p-value < 0.001); trusting CMDs for health advice was associated with an additional 0.048 (p-value = 0.001) probability of compliance (Table 1, Table 2). In the analysis of directed network connections, compliance only was positively associated (p-value < 0.05) with an individual naming a CMD as someone trusted for medical advice (Supplementary Tables 6-7). Individuals whom CMDs turned to for health advice were not more likely (p-value > 0.05) to ingest medicines when offered treatment by CMDs (Supplementary Tables 6-7). Being close friends with CMDs was weakly correlated with trusting CMDs for health advice (Obs. 14431, Spearman ρ 0.214, p-value < 0.001). However, having a connection to CMDs in the friendship network did not increase compliance (p-value > 0.05). Lastly, formal social status (Table 1, Table 2) was not related to compliance.

## 4 Discussion

Social biases of CMDs prevented MDA from working as intended by the national control programme. CMDs offered treatment to people with powerful network positions. Individuals with high friendship degree and high friendship closeness centrality were most likely to be offered not only at least one drug during MDA, but also to be administered the correct combination of pills. Moreover, social inequalities in treatment, e.g. variation in socioeconomic status, manifested in the CMDs' decisions to distribute treatment and not in the individual's decision to accept the drugs offered. The treatment of individuals with high centrality aided CMDs in gaining social status. Accordingly, exploiting the social biases of CMDs could improve MDA implementation. Volunteer CMDs, who incur high opportunity costs (Fleming et al., 2016, Katabarwa et al., 2010b), can be incentivized without monetary payments. Providing recognition to CMDs for treating disadvantaged groups from individuals with more social resources, i.e. village influence, can assist CMDs in constructing upward social ties in the village network for attaining or retaining their personal status whilst achieving objectives of MDA (Lin et al., 1981).

Though distributing drugs implies providing a (local) public good, our findings showed that CMDs were not primarily motivated by a desire for prosocial behavior or altruism (Ariely et al., 2009, Bénabou and Tirole, 2006). If CMDs were behaving prosocially, i.e. trying to appear fair and benevolent to most community members, so as to create a positive image that boosts CMD social status, then they would have treated the most socioeconomically disadvantaged households. Similarly, CMDs did not strive for MDA efficiency during drug distribution, as they would have targeted less privileged individuals who are suspected to harbour the heaviest infection intensities.

Approaching influential individuals provided CMDs with the visibility, status, and social acceptability that otherwise would be received from treating the wider community. Instead of approaching all eligible individuals, CMDs treated the well-connected or well-embedded people in the community. These treated individuals directly observed the CMDs conducting MDA. In turn, CMDs appeared benevolent to powerful members of the community, i.e. individuals with influential network positions. These influential individuals might then transfer their perception of CMD benevolence, importance, and rank to other village members. If this diffusion was occurring, CMDs were efficient self-promoters who directly or unintentionally targeted the individuals who had the most connections (friendship degree) and best access (friendship closeness) to other community members (Rogers, 2003). Additionally, influential individuals, when interacting with CMDs, could also be perceived by the community to be associated with CMDs and in turn confer status to CMDs (Kilduff and Krackhardt, 1994). Notably, beyond the image of the CMD, there was no evidence that people with high friendship degree and closeness, were spreading actual information about MDA since untreated individuals often do not know that drugs are available (Chami et al., 2016). As the number of people with high network or social status is limited, future work should explore how CMDs choose whom to treat amongst the people without status. Though nonlinear, the positive association of friendship closeness with coverage may suggest that CMDs first approach individuals based on status then put minimal effort to find other individuals in the social network, i.e. by following the shortest network paths.

The pursuit of status and recognition from peers—especially powerful individuals—is consistent with the hypothesis that CMDs distribute pills because it is in their self-interests. Accordingly, we anticipate that our findings will extend to other community-directed MDA programmes either elsewhere in Uganda, in other sub-Saharan countries, or for different infections such as onchocerciasis, lymphatic filariasis, and trachoma. Moreover, any public intervention through CMDs, such as bed net distribution, might be affected by CMD social biases. Issues to consider for other contexts include the impact of payment on CMD motivations (beyond training remuneration), the frequency of CMD turnover if CMDs cannot retain their position long enough to gain village status, and ensuring that close friendship is defined in locally relevant terms. Ideally, social biases would not be applicable for school-based MDA. However, this conjecture depends on school-based MDA working as intended, i.e. schoolteachers simply treating all children in attendance. There is a need to examine if primary school teachers, who also volunteer in MDA, directly or unintentionally provide discriminatory treatment.

Across villages, the level of compliance may have been related to the level of coverage due to observed and unobserved factors. Trust in CMDs was associated with both coverage and compliance and might be a directly targetable aspect of CMD behavior for increasing treatment. Unobserved factors may include treatment spillover effects where individuals are more likely to comply with treatment when a greater proportion of their community is offered treatment. Here we focused on systematic noncompliance and programme implementation. However, future studies concerned with individual take-up of MDA will want to consider exactly which drugs were refused when offered.

Two strategies could be employed alongside routine training and MDA implementation. First, national programs should instruct individuals responsible for training and overseeing CMDs to monitor the treatment of households that do not have many friends in the village. Such monitoring is possible as we showed that both CMDs and the general population widely and easily recognized high friendship degree (Supplementary Data). Second, make all treatment offers public events. Currently, drugs are distributed in private; most individuals who are not offered treatment do not know that any drugs are available in the village (Chami et al., 2016). Public charts could be used in each village to record the households offered treatment. This method of increasing the visibility of drug distribution may redirect CMDs to act in a more prosocial manner in order to gain social status (Ariely et al., 2009, Bénabou and Tirole, 2006) and, importantly, improve treatment coverage.

Though the lack of health education has been described elsewhere as a major reason for noncompliance in MDA (Krentel et al., 2013), we also found a deeper issue at stake. Most individuals did not trust CMDs for health advice. CMDs were responsible for conducting health education about MDA in their village. If only the reason for noncompliance were examined without the analysis of health networks, an intervention to increase the number or intensity of health campaigns would be advocated. However, such an intervention alone is insufficient if noncompliers and the majority of villagers do not trust CMDs. Future health education campaigns should involve respected members of the community, such as local council members or popular individuals with many friends to build trust for health advice between CMDs and the recipients of MDA. Local council members and well-connected individuals already were more likely to be offered treatment by CMDs, so what is needed is for these individuals to convince their contacts to trust CMDs for health advice. Future work may investigate the use of communitywide nominations of who is most trusted for health advice. This information could be used to find individuals to include in sensitization campaigns. National control programmes also may provide CMDs with the list of individuals who are widely trusted for health advice. CMDs could be encouraged to treat these individuals since they were not more likely to be offered medicines when compared to people who were not widely trusted for health advice. Utilizing social networks to motivate CMDs and ensure they know the importance of treating the wider community could increase coverage of, and compliance with, mass treatment in developing countries.
