# Supplementary material for: Community-directed mass drug administration is undermined by status seeking in friendship networks and inadequate trust in health advice networks
Source: Soc Sci Med. 2017 Jun;183:37–47. doi: 10.1016/j.socscimed.2017.04.009 (PMC5446315; doi:10.1016/j.socscimed.2017.04.009)
Supplement: Supplementary material [file mmc1.docx]

**Supplementary information for:** Community-directed mass drug administration is undermined by status seeking in friendship networks and inadequate trust in health advice networks

Table of Contents

Supplementary data 2

Figure 1: Locally weighted regression (LOWESS) plots 3

Figure 2: Treatment outcomes for households in 17 study villages 4

Table 1: Univariate regressions against dependent variable of coverage 5

Table 2: Centrality spearman correlations 6

Table 3: Descriptive statistics 7

Table 4: Descriptive statistics continued 8

Table 5: Test of differences between people with and without treatment outcomes 9

Table 6: Average marginal effects (AMEs) of treatment probability for directed network indicators in friendship degree model 10

Table 7: AMEs of treatment probability for directed network indicators in friendship closeness centrality model 11

Table 8: AMEs from bivariate probit model with friendship degree 12

Table 9: AMEs from bivariate probit model with friendship closeness 13

Table 10: AMEs from bivariate probit model with friendship degree amongst only individuals offered at least one drug by CMDs 14

Table 11: AMEs from bivariate probit model with friendship closeness amongst only individuals offered at least one drug by CMDs 15

Table 12: Two-sample t-tests for centrality against formal social status 16

Table 13: Relationship with most trusted person for health advice 17

## Supplementary data

**Focus groups**

In April 2016, analyses of community medicine distributor (CMD) behaviors were completed through focus groups. The 34 CMDs from the 17 study villages were organized into six separate focus groups based on geographical proximity. This group included 4/34 new CMDs, who were not the drug distributors in 2013. CMDs were asked: “Why do you volunteer to be a drug distributor? What are the benefits to you?” Though CMDs in three villages also provided reasons concerning the ability to help others get well, the main reasons voiced by all for why individuals chose to be a CMD were 1) to gain preferential health treatment and 2) to gain social status. Concerning preferential treatment, one CMD stated “[I am] given first priority when [I] go to [the] health facility. Even if don’t tell them then they see the CMD shirt and treat me like a coworker.” Another CMD explained, “even for other malaria drugs, [we] can get treatment first in health centre[s].” A different CMD noted she “gets access to medicine and any person who is sick can communicate through her to tell the hospital to give them the medicine.” These statements were common and reiterated in every focus group.

Secondly, individuals chose to become CMDs to gain social status within their village. Two individuals from our study were able to become chairmen (leaders of their village governments) because they were first CMDs. Another CMD stated that social status was gained because they can “get friendship and can interact with higher people.” Several CMDs enthusiastically explained “cmds are very high social status in the community and even call them doctors in the village.” More comments on social status from many CMDs were provided. Additional examples include “[I am] able to communicate to the health workers and higher people; [I am] able to be known in the community”; “seeking to be recognized to meet big people…”; and “to get friendships [sic] from the community and other people in the broader community.” Unfortunately, such social status may preclude CMDs from interacting with less privileged individuals who are mostly likely to be infected and need treatment, due to poor sanitation. One CMD requested and five others agreed (in three villages) that she “needs a rain coat and gum boots, [because] some homes are very dirty and [she] need[s] protection.”

**Friendship degree visibility**

*Most close friends guesstimate networks prompt:* Only one guess was permitted and the prompt was presented immediately after the friendship network survey question.

“Please tell me the clan name first then the second name of who you think has the most close friends in this village.”

Twenty-nine different households were nominated by 30/34 CMDs. The remainder of the study population (3152 respondents) suggested 859 unique households. With only one nomination, CMDs and the general population together identified people in the 90^th^ percentile of friendship degree with 64.80% accuracy. Furthermore, considering the frequency of correct guesses, there was no significant difference between the ability of CMDs (60%, 18/30) and the general population (64.85%, 2044/3152) to recognize high friendship degree (Chi^2^ 0.306, p-value=0.580).

## Figure 1: Locally weighted regression (LOWESS) plots

## Figure 2: Treatment outcomes for households in 17 study villages


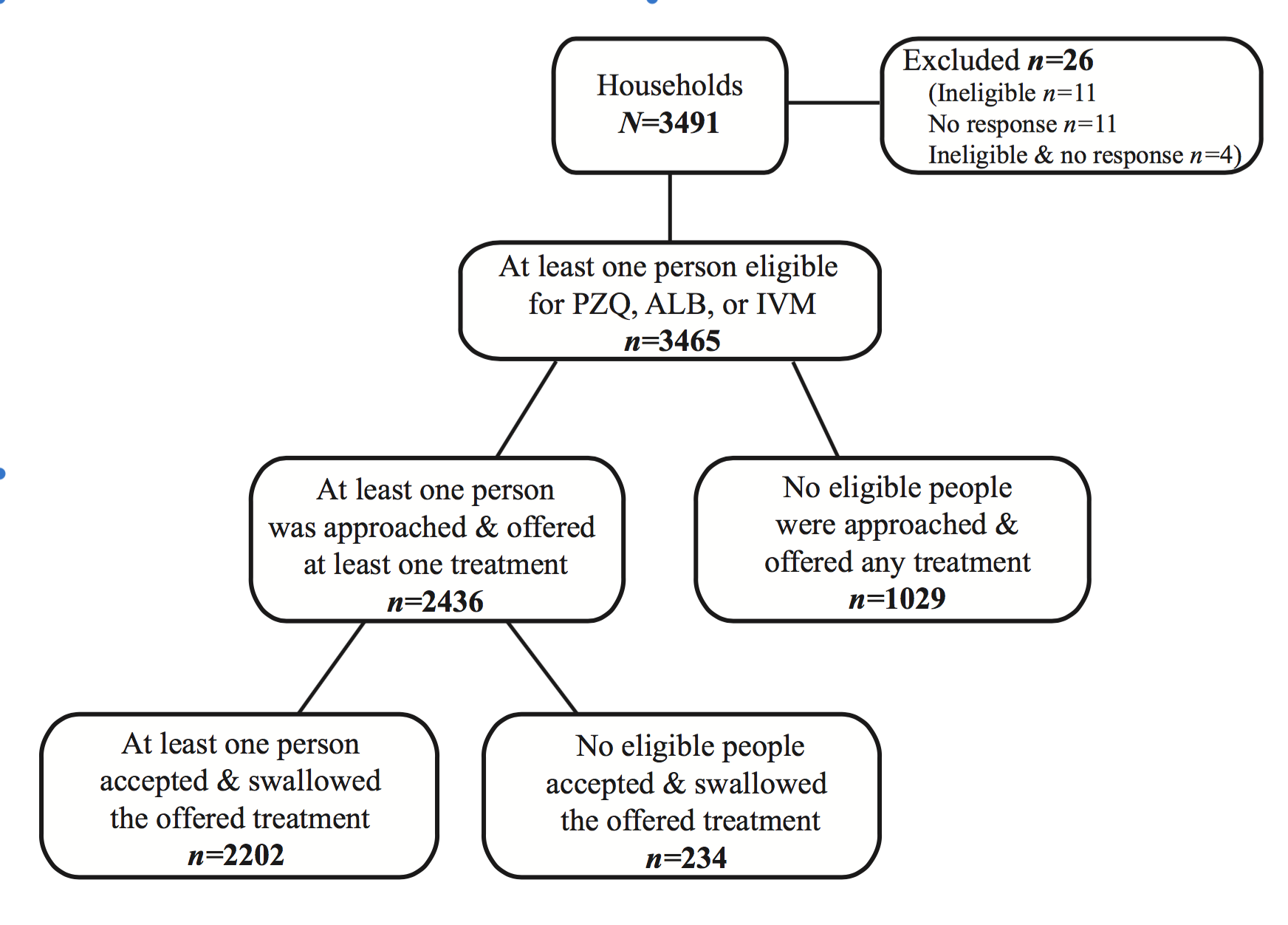


The distribution of praziquantel (PZQ), albendazole (ALB), or ivermectin (IVM) is provided for households with at least one eligible person. Households with no eligible people for mass drug administration had only individuals with severe illness or pregnant women (first trimester for IVM). No responses included households with individuals who chose not to provide a response, but also mostly individuals who did not remember being offered treatment.

## Table 1: Univariate regressions against dependent variable of coverage

| **Univariate Probit regressions, separate models** *constants not shown* | **Variables** | **Coeff.** | **Clustered Std. Err.** | **p-value** | **95% CI** | |
| --- | --- | --- | --- | --- | --- | --- |
| 1) | LN(Friendship degree +1), [0, 3.526] | 0.117 | 0.033 | <0.001 | 0.053 | 0.181 |
|  | LN(Friendship degree +1), [>3.526] | -0.313 | 0.645 | 0.628 | -1.578 | 0.952 |
| 2) | LN(Health degree +1) | 0.004 | 0.029 | 0.902 | -0.053 | 0.060 |
|  | (LN(Health degree +1))^2^ | 0.027 | 0.023 | 0.234 | -0.017 | 0.071 |
| 3) | LN(Health degree+1) | 0.012 | 0.029 | 0.692 | -0.046 | 0.069 |
| 4) | Friendship closeness centrality | 2.279 | 0.338 | <0.001 | 1.616 | 2.942 |
|  | Friendship closeness centrality^2^ | 4.931 | 1.367 | <0.001 | 2.252 | 7.611 |
| 5) | Friendship betweenness centrality | 8.734 | 2.019 | <0.001 | 4.775 | 12.693 |
|  | Friendship betweenness centrality^2^ | -61.976 | 18.547 | 0.001 | -98.342 | -25.611 |
| 6) | Health betweenness centrality [0, 0.268] | 2.446 | 0.734 | 0.001 | 1.006 | 3.886 |
|  | Health betweenness centrality [>0.268] | -3.920 | 2.615 | 0.134 | -9.048 | 1.208 |
| 7) | Close friends with CMD | 0.118 | 0.049 | 0.016 | 0.022 | 0.214 |
| 8) | Trusts CMD for health advice | 0.218 | 0.042 | <0.001 | 0.135 | 0.302 |
| Obs. | 14431 |  |  |  |  |  |
| Households | 3387 |  |  |  |  |  |

## Table 2: Centrality spearman correlations

|  | LN(Friendship degree +1) | LN(Health degree +1) | Friendship betweenness | Health betweenness | Friendship closeness | Health closeness |
| --- | --- | --- | --- | --- | --- | --- |
| LN(Friendship degree +1) | 1 |  |  |  |  |  |
| LN(Health degree +1) | 0.642* | 1 |  |  |  |  |
| Friendship betweenness | 0.779* | 0.461* | 1 |  |  |  |
| Health betweenness | 0.513* | 0.802* | 0.523* | 1 |  |  |
| Friendship closeness | 0.814* | 0.512* | 0.597* | 0.411* | 1 |  |
| Health closeness | 0.412* | 0.613* | 0.309* | 0.487* | 0.496* | 1 |
| Obs. 14431 |  |  |  |  |  |  |
| *p-value<0.05 |  |  |  |  |  |  |

## Table 3: Descriptive statistics

|  | ***A) Population eligible for treatment*** | | | ***B) Eligible individuals offered treatment*** | | | ***C) Eligible individuals not offered treatment*** | | |
| --- | --- | --- | --- | --- | --- | --- | --- | --- | --- |
| **Descriptive variables** | **Obs.** | **Mean *Ratio*** | **Std. Dev. *Freq.*** | **Obs.** | **Mean *Ratio*** | **Std. Dev. *Freq.*** | **Obs.** | **Mean**  ***Ratio*** | **Std. Dev. *Freq.*** |
| Coverage | 14754 | *0.567* | *8359* | - | *-* | *-* | - | *-* | *-* |
| Compliance | - | - | - | 8359 | *0.886* | *7405* | - | *-* | *-* |
| Friendship degree | 14754 | 10.675 | 8.120 | 8359 | 11.039 | 8.330 | 6395 | 10.199 | 7.811 |
| Health degree | 14754 | 6.590 | 10.003 | 8359 | 6.866 | 11.103 | 6395 | 6.229 | 8.337 |
| Friendship betweenness | 14754 | 0.012 | 0.020 | 8359 | 0.012 | 0.020 | 6395 | 0.011 | 0.021 |
| Health betweenness | 14754 | 0.014 | 0.047 | 8359 | 0.016 | 0.052 | 6395 | 0.012 | 0.040 |
| Friendship closeness | 14754 | 0.383 | 0.069 | 8359 | 0.389 | 0.069 | 6395 | 0.374 | 0.068 |
| Health closeness | 14754 | 0.338 | 0.097 | 8359 | 0.345 | 0.099 | 6395 | 0.329 | 0.094 |
| Close friends with a CMD | 14754 | *0.275* | *4062* | 8359 | *0.293* | *2446* | 6395 | *0.253* | *1616* |
| Trusts a CMD for health advice | 14754 | *0.414* | *6105* | 8359 | *0.451* | *3771* | 6395 | *0.365* | *2334* |
| Age in years | 14754 | 20.110 | 16.698 | 8359 | 20.433 | 17.003 | 6395 | 19.689 | 16.283 |
| Female | 14754 | *0.499* | *7367* | 8359 | *0.506* | *4231* | 6395 | *0.490* | *3136* |
| Social status: was or is a religious, tribe, or clan leader or on the local village council | 14754 | *0.029* | *426* | 8359 | *0.034* | *284* | 6395 | *0.022* | *142* |
| Fisherman or fishmonger | 14754 | *0.072* | *1067* | 8359 | *0.067* | *557* | 6395 | *0.080* | *510* |
| Other income-earning occupation | 14754 | *0.219* | *3233* | 8359 | *0.227* | *1900* | 6395 | *0.208* | *1333* |
| Highest level of education attained | 14754 | 3.484 | 3.300 | 8359 | 3.343 | 3.163 | 6395 | 3.669 | 3.462 |
| Muslim household head | 14753 | *0.301* | *4440* | 8358 | *0.284* | *2374* | 6395 | *0.323* | *2066* |
| Household head belongs to village majority tribe | 14506 | *0.396* | *5742* | 8230 | *0.409* | *3368* | 6276 | *0.378* | *2374* |
| No home latrine | 14754 | *0.085* | *1251* | 8359 | *0.069* | *578* | 6395 | *0.105* | *673* |
| Home owned, not rented | 14678 | *0.897* | *13161* | 8318 | *0.906* | *7538* | 6360 | *0.884* | *5623* |
| Home quality score | 14754 | 6.975 | 3.385 | 8359 | 7.105 | 3.334 | 6395 | 6.804 | 3.443 |

Two village level variables were used in the main analyses (Obs. 17)—total homes in the village (Avg. 210.471, Std. dev. 88.49) and an indicator for if the village centre was more than 0.50 km from Lake Victoria (Obs. 14754, 57.1% of all eligible individuals; Frequency 8,419).

## Table 4: Descriptive statistics continued

|  | ***A) Eligible individuals offered treatment & not complying*** | | | ***B) Eligible individuals offered treatment & complying*** | | |
| --- | --- | --- | --- | --- | --- | --- |
| **Descriptive variables** | **Obs.** | **Mean *Ratio*** | **Std. Dev. *Freq.*** | **Obs.** | **Mean *Ratio*** | **Std. Dev. *Freq.*** |
| Coverage | - | *-* | *-* | - | *-* | *-* |
| Compliance | - | - | - | - | *-* | *-* |
| Friendship degree | 954 | 10.676 | 7.919 | 7405 | 11.086 | 8.381 |
| Health degree | 954 | 6.310 | 8.545 | 7405 | 6.938 | 11.389 |
| Friendship betweenness | 954 | 0.010 | 0.015 | 7405 | 0.013 | 0.020 |
| Health betweenness | 954 | 0.009 | 0.024 | 7405 | 0.017 | 0.055 |
| Friendship closeness | 954 | 0.378 | 0.070 | 7405 | 0.390 | 0.069 |
| Health closeness | 954 | 0.338 | 0.085 | 7405 | 0.346 | 0.100 |
| Close friends with a CMD | 954 | *0.224* | *214* | 7405 | *0.301* | *2232* |
| Trusts a CMD for health advice | 954 | *0.339* | *323* | 7405 | *0.466* | *3448* |
| Age in years | 954 | 20.125 | 17.086 | 7405 | 20.473 | 16.993 |
| Female | 954 | *0.472* | *450* | 7405 | *0.511* | *3781* |
| Social status: was or is a religious, tribe, or clan leader or on the local village council | 954 | *0.028* | *27* | 7405 | *0.035* | *257* |
| Fisherman or fishmonger | 954 | *0.088* | *84* | 7405 | *0.064* | *473* |
| Other income-earning occupation | 954 | *0.199* | *190* | 7405 | *0.231* | *1710* |
| Highest level of education attained | 954 | 3.730 | 3.365 | 7405 | 3.293 | 3.133 |
| Muslim household head | 954 | *0.309* | *295* | 7404 | *0.281* | *2079* |
| Household head belongs to village majority tribe | 940 | *0.334* | *314* | 7290 | *0.419* | *3054* |
| No home latrine | 954 | *0.083* | *79* | 7405 | *0.067* | *499* |
| Home owned, not rented | 948 | *0.895* | *848* | 7370 | *0.908* | *6690* |
| Home quality score | 954 | 6.965 | 3.611 | 7405 | 7.123 | 3.297 |

## Table 5: Test of differences between people with and without treatment outcomes

|  |  | ***Eligible individuals classified in treatment outcomes*** | | | ***Eligible individuals not classified in treatment outcomes*** | | |  |  |  |
| --- | --- | --- | --- | --- | --- | --- | --- | --- | --- | --- |
|  | **Variable** | **Obs.** | **Mean** | **Std. Err.** | **Obs.** | **Mean** | **Std. Err.** | **t** | **p-value** | **df** |
| **Two sample t-tests** | Friendship degree | 14754 | 10.675 | 0.067 | 788 | 9.605 | 0.244 | 3.629 | <0.001 | 15540 |
|  | Health degree | 14754 | 6.590 | 0.082 | 788 | 6.176 | 0.337 | 1.134 | 0.257 | 15540 |
|  | Friendship betweenness | 14754 | 0.012 | <0.001 | 788 | 0.014 | 0.001 | -2.716 | 0.007 | 15540 |
|  | Health betweenness | 14754 | 0.014 | <0.001 | 788 | 0.016 | 0.002 | -1.130 | 0.259 | 15540 |
|  | Friendship closeness | 14754 | 0.383 | 0.001 | 788 | 0.373 | 0.003 | 3.752 | <0.001 | 15540 |
|  | Health closeness | 14754 | 0.338 | 0.001 | 788 | 0.329 | 0.004 | 2.471 | 0.014 | 15540 |
|  | Age | 14754 | 20.110 | 0.137 | 788 | 17.272 | 0.573 | 4.659 | <0.001 | 15540 |
|  | Highest level of education attained | 14754 | 3.484 | 0.027 | 788 | 3.253 | 0.127 | 1.911 | 0.056 | 15540 |
|  | Home quality score | 14754 | 6.975 | 0.028 | 788 | 7.133 | 0.124 | -1.280 | 0.201 | 15540 |
|  |  |  |  |  |  |  |  | **z** |  |  |
| **Two sample test of proportions** | Close friends with a CMD | 14754 | 0.275 | 0.004 | 788 | 0.310 | 0.016 | -2.098 | 0.036 | - |
|  | Trusts a CMD for health advice | 14754 | 0.414 | 0.004 | 788 | 0.392 | 0.017 | 1.203 | 0.229 | - |
|  | Female | 14754 | 0.499 | 0.004 | 788 | 0.470 | 0.018 | 1.629 | 0.103 | - |
|  | Social status: was or is a religious, tribe, or clan leader or on the local village council | 14754 | 0.029 | 0.001 | 788 | 0.023 | 0.005 | 0.990 | 0.322 | - |
|  | Fisherman or fishmonger | 14754 | 0.072 | 0.002 | 788 | 0.055 | 0.008 | 1.885 | 0.059 | - |
|  | Other income-earning occupation | 14754 | 0.219 | 0.003 | 788 | 0.194 | 0.014 | 1.654 | 0.098 | - |
|  | Muslim household head | 14753 | 0.301 | 0.004 | 788 | 0.349 | 0.017 | -2.857 | 0.004 | - |
|  | Household head belongs to village majority tribe | 14506 | 0.396 | 0.004 | 742 | 0.384 | 0.018 | 0.638 | 0.524 | - |
|  | No home latrine | 14754 | 0.085 | 0.002 | 788 | 0.051 | 0.008 | 3.372 | 0.001 | - |
|  | Home owned, not rented | 14678 | 0.897 | 0.003 | 783 | 0.880 | 0.012 | 1.490 | 0.136 | - |

## Table 6: Average marginal effects (AMEs) of treatment probability for directed network indicators in friendship degree model

|  | ***A) First stage dependent variable: coverage*** | | | | | ***B) Second stage dependent variable: compliance*** | | | | | |
| --- | --- | --- | --- | --- | --- | --- | --- | --- | --- | --- | --- |
| **Predictors** | **AME** | **Clustered Std. Err.** | **p-value** | **95% CI** | | **AME \| Coverage=1** ^a^ | **Clustered Std. Err.** | **p-value** | **95% CI** | |  |
| LN(Friendship in-degree +1) | 0.060 | 0.013 | <0.001 | 0.035 | 0.085 |  |  |  |  |  |  |
| Friendship out-degree^b^ | 0.013 | 0.003 | <0.001 | 0.007 | 0.020 |  |  |  |  |  |  |
| Friendship betweenness centrality ^b^ | -1.521 | 1.055 | 0.149 | -3.590 | 0.547 |  |  |  |  |  |  |
| Health betweenness centrality not equal to 0 | -0.090 | 0.017 | <0.001 | -0.125 | -0.056 |  |  |  |  |  |  |
| CMD named household as friend (outgoing edge from CMD) | 0.009 | 0.034 | 0.788 | -0.058 | 0.076 | 0.003 | 0.032 | 0.932 | -0.061 | 0.066 |  |
| Household named CMD as friend (incoming edge to CMD) | -0.007 | 0.021 | 0.730 | -0.048 | 0.034 | 0.029 | 0.019 | 0.123 | -0.008 | 0.065 |  |
| CMD trusted household for health advice (outgoing edge from CMD) | 0.068 | 0.048 | 0.153 | -0.025 | 0.162 | 0.050 | 0.037 | 0.180 | -0.023 | 0.124 |  |
| Household trusted CMD for health advice (incoming edge to CMD) | 0.065 | 0.016 | <0.001 | 0.033 | 0.096 | 0.043 | 0.015 | 0.004 | 0.013 | 0.072 |  |
| Age in years | 0.001 | <0.001 | 0.003 | <0.001 | 0.002 | <0.001 | <0.001 | 0.557 | <-0.001 | 0.001 |  |
| Female | 0.011 | 0.008 | 0.169 | -0.005 | 0.027 | 0.011 | 0.008 | 0.151 | -0.004 | 0.026 |  |
| Social status: was or is a religious, tribe, or clan leader or on the local village council | 0.078 | 0.026 | 0.003 | 0.027 | 0.130 | 0.019 | 0.025 | 0.436 | -0.029 | 0.068 |  |
| Fisherman or fishmonger | -0.017 | 0.019 | 0.357 | -0.054 | 0.020 | -0.002 | 0.016 | 0.902 | -0.034 | 0.030 |  |
| Other income-earning occupation | 0.018 | 0.013 | 0.181 | -0.008 | 0.044 | 0.028 | 0.013 | 0.028 | 0.003 | 0.052 |  |
| Highest level of education attained | -0.009 | 0.002 | <0.001 | -0.012 | -0.006 | -0.006 | 0.002 | <0.001 | -0.009 | -0.003 |  |
| Muslim household head | -0.049 | 0.017 | 0.004 | -0.083 | -0.015 | -0.022 | 0.015 | 0.135 | -0.051 | 0.007 |  |
| Household head belongs to village majority tribe | 0.026 | 0.016 | 0.104 | -0.005 | 0.057 | 0.037 | 0.014 | 0.009 | 0.009 | 0.065 |  |
| No home latrine | -0.085 | 0.028 | 0.002 | -0.140 | -0.031 | <-0.001 | 0.024 | 0.995 | -0.048 | 0.047 |  |
| Home owned, not rented | 0.013 | 0.024 | 0.600 | -0.035 | 0.060 | 0.003 | 0.023 | 0.913 | -0.043 | 0.048 |  |
| Home quality score | 0.002 | 0.002 | 0.440 | -0.003 | 0.007 | -0.001 | 0.002 | 0.770 | -0.005 | 0.004 |  |
| Village centre greater than 0.50 km from Lake Victoria | -0.150 | 0.017 | <0.001 | -0.182 | -0.117 |  |  |  |  |  |  |
| Total homes in village | -0.001 | <0.001 | <0.001 | -0.001 | -0.001 |  |  |  |  |  |  |
| Obs. 14431 |  |  |  |  |  |  |  |  |  |  |  |
| Households 3387 |  |  |  |  |  |  |  |  |  |  |  |
| Inverse hyperbolic tangent of 𝜌 | -1.286 | 0.357 | <0.001 | -1.985 | -0.586 |  |  |  |  |  |  |
| 𝜌 | -0.858 | 0.094 |  | -0.963 | -0.527 |  |  |  |  |  |  |
| Wald test of independent equations, Chi^2^ | 12.97 |  | <0.001 |  |  |  |  |  |  |  |  |

^a^ Average marginal probability of compliance =1 conditional on coverage (being offered treatment)

^b^ Entered in this Heckman selection model as grand-mean centered and a quadratic, i.e. also including the squared form. All covariate functional forms were determined as described in the main text Methods.

## Table 7: AMEs of treatment probability for directed network indicators in friendship closeness centrality model

|  | ***A) First stage dependent variable: coverage*** | | | | | ***B) Second stage dependent variable: compliance*** | | | | | | |
| --- | --- | --- | --- | --- | --- | --- | --- | --- | --- | --- | --- | --- |
| **Predictors** | **AME** | **Clustered Std. Err.** | **p-value** | **95% CI** | | **AME \| Coverage=1**^a^ | **Clustered Std. Err.** | **p-value** | **95% CI** | |  |  |
| Friendship closeness centrality ^b^ | 1.308 | 0.158 | <0.001 | 0.998 | 1.618 |  |  |  |  |  | |  |
| Friendship betweenness centrality ^b^ | 0.948 | 0.817 | 0.246 | -0.653 | 2.550 |  |  |  |  |  | |  |
| Health betweenness centrality not equal to 0 | -0.072 | 0.017 | <0.001 | -0.105 | -0.040 |  |  |  |  |  | |  |
| CMD named household as friend (outgoing edge from CMD) | 0.020 | 0.033 | 0.554 | -0.046 | 0.085 | 0.006 | 0.032 | 0.852 | -0.057 | 0.069 | |  |
| Household named CMD as friend (incoming edge to CMD) | -0.022 | 0.020 | 0.273 | -0.062 | 0.018 | 0.026 | 0.019 | 0.171 | -0.011 | 0.062 | |  |
| CMD trusted household for health advice (outgoing edge from CMD) | 0.074 | 0.048 | 0.123 | -0.020 | 0.168 | 0.053 | 0.037 | 0.154 | -0.020 | 0.126 | |  |
| Household trusted CMD for health advice (incoming edge to CMD) | 0.065 | 0.016 | <0.001 | 0.034 | 0.097 | 0.044 | 0.015 | 0.004 | 0.014 | 0.074 | |  |
| Age in years | <0.001 | <0.001 | 0.005 | <0.001 | 0.002 | <0.001 | <0.001 | 0.553 | <-0.001 | 0.001 | |  |
| Female | 0.010 | 0.008 | 0.193 | -0.005 | 0.026 | 0.011 | 0.008 | 0.160 | -0.004 | 0.026 | |  |
| Social status: was or is a religious, tribe, or clan leader or on the local village council | 0.084 | 0.027 | 0.002 | 0.032 | 0.136 | 0.022 | 0.025 | 0.383 | -0.027 | 0.071 | |  |
| Fisherman or fishmonger | -0.019 | 0.019 | 0.306 | -0.056 | 0.017 | -0.003 | 0.016 | 0.847 | -0.035 | 0.029 | |  |
| Other income-earning occupation | 0.011 | 0.013 | 0.412 | -0.015 | 0.037 | 0.027 | 0.013 | 0.036 | 0.002 | 0.051 | |  |
| Highest level of education attained | -0.008 | 0.002 | <0.001 | -0.012 | -0.005 | -0.006 | 0.002 | <0.001 | -0.009 | -0.003 | |  |
| Muslim household head | -0.045 | 0.017 | 0.007 | -0.078 | -0.012 | -0.022 | 0.015 | 0.145 | -0.051 | 0.007 | |  |
| Household head belongs to village majority tribe | 0.033 | 0.016 | 0.037 | 0.002 | 0.064 | 0.039 | 0.014 | 0.006 | 0.011 | 0.067 | |  |
| No home latrine | -0.088 | 0.028 | 0.002 | -0.142 | -0.033 | -0.002 | 0.025 | 0.944 | -0.050 | 0.047 | |  |
| Home owned, not rented | 0.025 | 0.024 | 0.296 | -0.022 | 0.072 | 0.005 | 0.023 | 0.842 | -0.041 | 0.051 | |  |
| Home quality score | 0.003 | 0.002 | 0.156 | -0.001 | 0.008 | <-0.001 | 0.002 | 0.861 | -0.005 | 0.004 | |  |
| Village centre greater than 0.50 km from Lake Victoria | -0.171 | 0.017 | <0.001 | -0.203 | -0.139 |  |  |  |  |  | |  |
| Total homes in village | -0.001 | <0.001 | <0.001 | -0.001 | <-0.001 |  |  |  |  |  | |  |
| Obs. 14431 |  |  |  |  |  |  |  |  |  |  | |  |
| Households 3387 |  |  |  |  |  |  |  |  |  |  | |  |
| Inverse hyperbolic tangent of 𝜌 | -1.293 | 0.328 | <0.001 | -1.936 | -0.651 |  |  |  |  |  | |  |
| 𝜌 | -0.860 | 0.085 |  | -0.959 | -0.572 |  |  |  |  |  | |  |
| Wald test of independent equations, Chi^2^ | 15.59 |  | <0.001 |  |  |  |  |  |  |  | |  |

^a^ Average marginal probability of compliance =1 conditional on coverage (being offered treatment)

^b^ Entered in this Heckman selection model as grand-mean centered and a quadratic, i.e. also including the squared form. All covariate functional forms were determined as described in the main text Methods.

## Table 8: AMEs from bivariate probit model with friendship degree

|  | ***A) Dependent variable: praziquantel*** | | | | | ***B) Dependent variable: albendazole & ivermectin*** | | | | | ***C) AMEs when both dependent variables (all drugs) =1*** | | | | |
| --- | --- | --- | --- | --- | --- | --- | --- | --- | --- | --- | --- | --- | --- | --- | --- |
| **Predictors** | **AME** | **Clustered Std. Err.** | **p-value** | **95% CI** | | **AME** | **Clustered Std. Err.** | **p-value** | **95% CI** | | **AME** | **Clustered Std. Err.** | **p-value** | **95% CI** | |
| LN(Friendship degree +1), [0, 3.526] | 0.092 | 0.016 | <0.001 | 0.060 | 0.124 | 0.109 | 0.016 | <0.001 | 0.078 | 0.139 | 0.099 | 0.014 | <0.001 | 0.071 | 0.127 |
| LN(Friendship degree +1), [>3.526] | 0.373 | 0.219 | 0.089 | -0.056 | 0.803 | 0.302 | 0.199 | 0.130 | -0.089 | 0.693 | 0.299 | 0.182 | 0.101 | -0.058 | 0.657 |
| Friendship betweenness centrality^a^ | -1.919 | 1.051 | 0.068 | -3.980 | 0.142 | -4.092 | 0.992 | <0.001 | -6.036 | -2.148 | -3.429 | 0.896 | <0.001 | -5.185 | -1.672 |
| Health betweenness centrality [0, 0.268] | 0.467 | 0.352 | 0.185 | -0.223 | 1.158 | 0.615 | 0.333 | 0.065 | -0.037 | 1.267 | 0.551 | 0.305 | 0.071 | -0.046 | 1.148 |
| Health betweenness centrality [>0.268] | 0.227 | 0.726 | 0.755 | -1.197 | 1.651 | 0.192 | 0.731 | 0.792 | -1.242 | 1.626 | 0.188 | 0.666 | 0.777 | -1.117 | 1.494 |
| Close friends with CMD | 0.005 | 0.018 | 0.804 | -0.031 | 0.040 | -0.009 | 0.018 | 0.594 | -0.044 | 0.025 | -0.006 | 0.016 | 0.702 | -0.037 | 0.025 |
| Trusts CMD for health advice | 0.074 | 0.015 | <0.001 | 0.044 | 0.104 | 0.069 | 0.015 | <0.001 | 0.040 | 0.098 | 0.066 | 0.013 | <0.001 | 0.040 | 0.092 |
| Age in years | 0.004 | <0.001 | <0.001 | 0.003 | 0.005 | 0.002 | <0.001 | <0.001 | 0.002 | 0.003 | 0.003 | <0.001 | <0.001 | 0.002 | 0.003 |
| Female | 0.014 | 0.008 | 0.082 | -0.002 | 0.031 | 0.020 | 0.008 | 0.008 | 0.005 | 0.035 | 0.018 | 0.007 | 0.008 | 0.005 | 0.031 |
| Social status: was or is a religious, tribe, or clan leader or on the local village council | 0.031 | 0.025 | 0.223 | -0.019 | 0.080 | 0.010 | 0.023 | 0.661 | -0.035 | 0.055 | 0.014 | 0.021 | 0.507 | -0.027 | 0.054 |
| Fisherman or fishmonger | -0.013 | 0.019 | 0.503 | -0.050 | 0.024 | 0.001 | 0.017 | 0.935 | -0.032 | 0.035 | -0.001 | 0.016 | 0.925 | -0.032 | 0.029 |
| Other income-earning occupation | -0.004 | 0.013 | 0.747 | -0.030 | 0.022 | -0.019 | 0.012 | 0.125 | -0.042 | 0.005 | -0.015 | 0.011 | 0.179 | -0.036 | 0.007 |
| Highest level of education attained | 0.006 | 0.002 | <0.001 | 0.003 | 0.009 | 0.005 | 0.001 | <0.001 | 0.003 | 0.008 | 0.005 | 0.001 | <0.001 | 0.003 | 0.008 |
| Muslim household head | -0.036 | 0.017 | 0.031 | -0.068 | -0.003 | -0.018 | 0.016 | 0.250 | -0.049 | 0.013 | -0.021 | 0.014 | 0.149 | -0.049 | 0.007 |
| Household head belongs to village majority tribe | 0.042 | 0.015 | 0.005 | 0.013 | 0.072 | 0.042 | 0.015 | 0.004 | 0.013 | 0.070 | 0.039 | 0.013 | 0.003 | 0.014 | 0.065 |
| No home latrine | -0.055 | 0.027 | 0.041 | -0.109 | -0.002 | -0.032 | 0.026 | 0.210 | -0.083 | 0.018 | -0.035 | 0.024 | 0.137 | -0.082 | 0.011 |
| Home owned, not rented | 0.033 | 0.022 | 0.129 | -0.010 | 0.076 | 0.027 | 0.021 | 0.200 | -0.014 | 0.067 | 0.026 | 0.019 | 0.155 | -0.010 | 0.063 |
| Home quality score | 0.002 | 0.002 | 0.350 | -0.002 | 0.006 | 0.003 | 0.002 | 0.178 | -0.001 | 0.007 | 0.003 | 0.002 | 0.185 | -0.001 | 0.006 |
| Village centre greater than 0.50 km from Lake Victoria | -0.135 | 0.016 | <0.001 | -0.168 | -0.103 | -0.107 | 0.016 | <0.001 | -0.137 | -0.076 | -0.106 | 0.014 | <0.001 | -0.134 | -0.078 |
| Total homes in village | -0.001 | <0.001 | <0.001 | -0.001 | -0.001 | -0.001 | <0.001 | <0.001 | -0.001 | -0.001 | -0.001 | <0.001 | <0.001 | -0.001 | -0.001 |
| Obs. 13496^b^ |  |  |  |  |  |  |  |  |  |  |  |  |  |  |  |
| Households 3283^b^ |  |  |  |  |  |  |  |  |  |  |  |  |  |  |  |
| Inverse hyperbolic tangent of 𝜌 | 1.442 | 0.040 | <0.001 | 1.364 | 1.519 |  |  |  |  |  |  |  |  |  |  |
| 𝜌 | 0.894 | 0.008 |  | 0.877 | 0.909 |  |  |  |  |  |  |  |  |  |  |
| Wald test of independent equations, Chi^2^ | 1275.86 |  | <0.001 |  |  |  |  |  |  |  |  |  |  |  |  |

^a^Entered in this bivariate probit model as grand-mean centered and a quadratic, i.e. also including the squared form. All covariate functional forms were determined using locally weighted regression ..

^b^Noncompliers were treated as missing.

## Table 9: AMEs from bivariate probit model with friendship closeness

|  | ***A) Dependent variable: praziquantel*** | | | | | ***B) Dependent variable: albendazole & ivermectin*** | | | | | ***C) AMEs when both dependent variables (all drugs) =1*** | | | | | | | |  |
| --- | --- | --- | --- | --- | --- | --- | --- | --- | --- | --- | --- | --- | --- | --- | --- | --- | --- | --- | --- |
| **Predictors** | **AME** | **Clustered Std. Err.** | **p-value** | **95% CI** | | **AME** | **Clustered Std. Err.** | **p-value** | **95% CI** | | **AME** | **Clustered Std. Err.** | | **p-value** | | **95% CI** | | |  |
| Friendship closeness centrality^a^ | 1.223 | 0.167 | <0.001 | 0.896 | 1.550 | 1.496 | 0.154 | <0.001 | 1.193 | 1.799 | 1.363 | | 0.142 | | <0.001 | | 1.084 | 1.642 | |
| Friendship betweenness centrality^a^ | -1.197 | 0.946 | 0.206 | -3.052 | 0.657 | -3.430 | 0.869 | <0.001 | -5.134 | -1.726 | -2.793 | | 0.786 | | <0.001 | | -4.334 | -1.252 | |
| Health betweenness centrality [0, 0.268] | 0.358 | 0.358 | 0.318 | -0.345 | 1.060 | 0.462 | 0.338 | 0.172 | -0.201 | 1.124 | 0.415 | | 0.310 | | 0.181 | | -0.193 | 1.023 | |
| Health betweenness centrality [>0.268] | 0.357 | 0.730 | 0.625 | -1.074 | 1.788 | 0.408 | 0.732 | 0.577 | -1.028 | 1.843 | 0.375 | | 0.667 | | 0.574 | | -0.932 | 1.682 | |
| Close friends with CMD | -0.018 | 0.019 | 0.333 | -0.055 | 0.019 | -0.038 | 0.018 | 0.035 | -0.073 | -0.003 | -0.032 | | 0.016 | | 0.050 | | -0.063 | <0.001 | |
| Trusts CMD for health advice | 0.072 | 0.015 | <0.001 | 0.043 | 0.102 | 0.066 | 0.014 | <0.001 | 0.038 | 0.094 | 0.064 | | 0.013 | | <0.001 | | 0.038 | 0.089 | |
| Age in years | 0.004 | <0.001 | <0.001 | 0.003 | 0.005 | 0.002 | <0.001 | <0.001 | 0.002 | 0.003 | 0.003 | | <0.001 | | <0.001 | | 0.002 | 0.003 | |
| Female | 0.014 | 0.008 | 0.091 | -0.002 | 0.030 | 0.020 | 0.008 | 0.009 | 0.005 | 0.035 | 0.018 | | 0.007 | | 0.010 | | 0.004 | 0.031 | |
| Social status: was or is a religious, tribe, or clan leader or on the local village council | 0.028 | 0.025 | 0.269 | -0.022 | 0.078 | 0.006 | 0.023 | 0.799 | -0.039 | 0.051 | 0.010 | | 0.021 | | 0.630 | | -0.030 | 0.050 | |
| Fisherman or fishmonger | -0.010 | 0.019 | 0.582 | -0.047 | 0.026 | 0.004 | 0.017 | 0.798 | -0.029 | 0.038 | 0.001 | | 0.015 | | 0.939 | | -0.029 | 0.031 | |
| Other income-earning occupation | -0.006 | 0.013 | 0.641 | -0.032 | 0.020 | -0.021 | 0.012 | 0.085 | -0.044 | 0.003 | -0.017 | | 0.011 | | 0.126 | | -0.038 | 0.005 | |
| Highest level of education attained | 0.006 | 0.002 | <0.001 | 0.003 | 0.009 | 0.005 | 0.001 | <0.001 | 0.003 | 0.008 | 0.005 | | 0.001 | | <0.001 | | 0.003 | 0.008 | |
| Muslim household head | -0.032 | 0.016 | 0.052 | -0.064 | <0.001 | -0.014 | 0.016 | 0.382 | -0.044 | 0.017 | -0.016 | | 0.014 | | 0.243 | | -0.044 | 0.011 | |
| Household head belongs to village majority tribe | 0.043 | 0.015 | 0.004 | 0.013 | 0.072 | 0.043 | 0.014 | 0.003 | 0.015 | 0.071 | 0.040 | | 0.013 | | 0.002 | | 0.015 | 0.066 | |
| No home latrine | -0.054 | 0.027 | 0.045 | -0.106 | -0.001 | -0.030 | 0.026 | 0.239 | -0.080 | 0.020 | -0.033 | | 0.023 | | 0.157 | | -0.079 | 0.013 | |
| Home owned, not rented | 0.041 | 0.022 | 0.058 | -0.001 | 0.084 | 0.036 | 0.021 | 0.080 | -0.004 | 0.076 | 0.035 | | 0.018 | | 0.057 | | -0.001 | 0.071 | |
| Home quality score | 0.003 | 0.002 | 0.239 | -0.002 | 0.007 | 0.004 | 0.002 | 0.095 | -0.001 | 0.008 | 0.003 | | 0.002 | | 0.099 | | -0.001 | 0.007 | |
| Village centre greater than 0.50 km from Lake Victoria | -0.143 | 0.016 | <0.001 | -0.175 | -0.111 | -0.118 | 0.015 | <0.001 | -0.148 | -0.087 | -0.116 | | 0.014 | | <0.001 | | -0.143 | -0.089 | |
| Total homes in village | <-0.001 | <0.001 | <0.001 | -0.001 | <0.001 | -0.001 | <0.001 | <0.001 | -0.001 | <-0.001 | <-0.001 | | <0.001 | | <0.001 | | -0.001 | <-0.001 | |
| Obs. 13496^b^ |  |  |  |  |  |  |  |  |  |  |  | |  | |  | |  |  | |
| Households 3283^b^ |  |  |  |  |  |  |  |  |  |  |  | |  | |  | |  |  | |
| Inverse hyperbolic tangent of 𝜌 | 1.439 | 0.040 | <0.001 | 1.361 | 1.517 |  |  |  |  |  |  | |  | |  | |  |  | |
| 𝜌 | 0.893 | 0.008 |  | 0.877 | 0.908 |  |  |  |  |  |  | |  | |  | |  |  | |
| Wald test of independent equations, Chi^2^ | 1263.88 |  | <0.001 |  |  |  |  |  |  |  |  | |  | |  | |  |  | |

^a^Entered in this bivariate probit model as grand-mean centered and a quadratic, i.e. also including the squared form. All covariate functional forms were determined using locally weighted regression.

^b^ Noncompliers were treated as missing.

## Table 10: AMEs from bivariate probit model with friendship degree amongst only individuals offered at least one drug by CMDs

|  | ***A) Dependent variable: praziquantel*** | | | | | ***B) Dependent variable: albendazole & ivermectin*** | | | | | ***C) AMEs when both dependent variables (all drugs) =1*** | | | | |
| --- | --- | --- | --- | --- | --- | --- | --- | --- | --- | --- | --- | --- | --- | --- | --- |
| **Predictors** | **AME** | **Clustered Std. Err.** | **p-value** | **95% CI** | | **AME** | **Clustered Std. Err.** | **p-value** | **95% CI** | | **AME** | **Clustered Std. Err.** | **p-value** | **95% CI** | |
| LN(Friendship degree +1), [0, 3.526] | 0.069 | 0.015 | <0.001 | 0.040 | 0.097 | 0.141 | 0.021 | <0.001 | 0.100 | 0.182 | 0.134 | 0.019 | <0.001 | 0.097 | 0.171 |
| LN(Friendship degree +1), [>3.526] | 0.225 | 0.214 | 0.292 | -0.194 | 0.644 | 0.110 | 0.278 | 0.692 | -0.436 | 0.656 | 0.157 | 0.244 | 0.521 | -0.323 | 0.636 |
| Friendship betweenness centrality^a^ | -1.349 | 0.922 | 0.144 | -3.157 | 0.459 | -6.779 | 1.361 | <0.001 | -9.447 | -4.111 | -5.857 | 1.187 | <0.001 | -8.185 | -3.529 |
| Health betweenness centrality [0, 0.268] | 0.165 | 0.339 | 0.626 | -0.499 | 0.829 | 0.718 | 0.477 | 0.132 | -0.217 | 1.653 | 0.629 | 0.445 | 0.157 | -0.243 | 1.502 |
| Health betweenness centrality [>0.268] | -0.057 | 0.723 | 0.938 | -1.474 | 1.361 | -0.065 | 0.995 | 0.948 | -2.015 | 1.886 | -0.069 | 0.932 | 0.941 | -1.896 | 1.758 |
| Close friends with CMD | 0.014 | 0.018 | 0.427 | -0.020 | 0.048 | -0.013 | 0.025 | 0.593 | -0.062 | 0.035 | -0.006 | 0.022 | 0.772 | -0.050 | 0.037 |
| Trusts CMD for health advice | 0.019 | 0.015 | 0.218 | -0.011 | 0.049 | 0.046 | 0.020 | 0.025 | 0.006 | 0.086 | 0.043 | 0.018 | 0.020 | 0.007 | 0.078 |
| Age in years | 0.006 | 0.001 | <0.001 | 0.005 | 0.007 | 0.004 | <0.001 | <0.001 | 0.003 | 0.004 | 0.005 | <0.001 | <0.001 | 0.004 | 0.006 |
| Female | -0.005 | 0.010 | 0.606 | -0.024 | 0.014 | 0.020 | 0.012 | 0.086 | -0.003 | 0.043 | 0.015 | 0.011 | 0.178 | -0.007 | 0.036 |
| Social status: was or is a religious, tribe, or clan leader or on the local village council | -0.053 | 0.033 | 0.116 | -0.118 | 0.013 | -0.071 | 0.034 | 0.036 | -0.137 | -0.005 | -0.073 | 0.031 | 0.017 | -0.133 | -0.013 |
| Fisherman or fishmonger | -0.031 | 0.025 | 0.220 | -0.081 | 0.019 | 0.009 | 0.027 | 0.746 | -0.044 | 0.062 | -0.002 | 0.025 | 0.926 | -0.051 | 0.046 |
| Other income-earning occupation | -0.067 | 0.018 | <0.001 | -0.102 | -0.033 | -0.076 | 0.018 | <0.001 | -0.112 | -0.040 | -0.082 | 0.017 | <0.001 | -0.115 | -0.049 |
| Highest level of education attained | 0.030 | 0.002 | <0.001 | 0.026 | 0.034 | 0.025 | 0.002 | <0.001 | 0.020 | 0.029 | 0.029 | 0.002 | <0.001 | 0.025 | 0.033 |
| Muslim household head | 0.014 | 0.017 | 0.410 | -0.019 | 0.046 | 0.027 | 0.023 | 0.244 | -0.018 | 0.071 | 0.026 | 0.021 | 0.213 | -0.015 | 0.066 |
| Household head belongs to village majority tribe | 0.017 | 0.015 | 0.270 | -0.013 | 0.047 | 0.033 | 0.020 | 0.100 | -0.006 | 0.072 | 0.032 | 0.018 | 0.077 | -0.003 | 0.067 |
| No home latrine | 0.013 | 0.027 | 0.642 | -0.040 | 0.065 | 0.030 | 0.037 | 0.407 | -0.041 | 0.102 | 0.028 | 0.033 | 0.395 | -0.037 | 0.094 |
| Home owned, not rented | 0.051 | 0.023 | 0.029 | 0.005 | 0.098 | 0.050 | 0.030 | 0.098 | -0.009 | 0.108 | 0.056 | 0.027 | 0.039 | 0.003 | 0.108 |
| Home quality score | 0.004 | 0.002 | 0.112 | -0.001 | 0.008 | 0.006 | 0.003 | 0.066 | 0.000 | 0.012 | 0.006 | 0.003 | 0.041 | <0.001 | 0.011 |
| Village centre greater than 0.50 km from Lake Victoria | -0.019 | 0.017 | 0.277 | -0.053 | 0.015 | -0.033 | 0.023 | 0.145 | -0.077 | 0.011 | -0.032 | 0.020 | 0.115 | -0.072 | 0.008 |
| Total homes in village | <-0.001 | <0.001 | 0.151 | <-0.001 | <0.001 | -0.001 | <0.001 | <0.001 | -0.001 | -0.001 | -0.001 | <0.001 | <0.001 | -0.001 | <-0.001 |
| Obs. 7254^b^ |  |  |  |  |  |  |  |  |  |  |  |  |  |  |  |
| Households 2159^b^ |  |  |  |  |  |  |  |  |  |  |  |  |  |  |  |
| Inverse hyperbolic tangent of 𝜌 | 0.728 | 0.040 | <0.001 | 0.650 | 0.806 |  |  |  |  |  |  |  |  |  |  |
| 𝜌 | 0.622 | 0.024 |  | 0.572 | 0.667 |  |  |  |  |  |  |  |  |  |  |
| Wald test of independent equations, Chi^2^ | 329.65 |  | <0.001 |  |  |  |  |  |  |  |  |  |  |  |  |

^a^Entered in this bivariate probit model as grand-mean centered and a quadratic, i.e. also including the squared form. All covariate functional forms were determined using locally weighted regression.

^b^ Noncompliers were treated as missing. Only individuals offered at least one drug were included.

## Table 11: AMEs from bivariate probit model with friendship closeness amongst only individuals offered at least one drug by CMDs

|  | ***A) Dependent variable: praziquantel*** | | | | | ***B) Dependent variable: albendazole & ivermectin*** | | | | | ***C) AMEs when both dependent variables (all drugs) =1*** | | | | |
| --- | --- | --- | --- | --- | --- | --- | --- | --- | --- | --- | --- | --- | --- | --- | --- |
| **Predictors** | **AME** | **Clustered Std. Err.** | **p-value** | **95% CI** | | **AME** | **Clustered Std. Err.** | **p-value** | **95% CI** | | **AME** | **Clustered Std. Err.** | **p-value** | **95% CI** | |
| Friendship closeness centrality ^a^ | 0.649 | 0.164 | <0.001 | 0.327 | 0.972 | 1.690 | 0.201 | <0.001 | 1.295 | 2.084 | 1.564 | 0.183 | <0.001 | 1.205 | 1.922 |
| Friendship betweenness centrality^a^ | -0.378 | 0.857 | 0.659 | -2.059 | 1.302 | -5.473 | 1.212 | <0.001 | -7.849 | -3.096 | -4.517 | 1.062 | <0.001 | -6.599 | -2.435 |
| Health betweenness centrality [0, 0.268] | 0.134 | 0.353 | 0.703 | -0.557 | 0.826 | 0.541 | 0.484 | 0.264 | -0.408 | 1.490 | 0.477 | 0.457 | 0.297 | -0.419 | 1.373 |
| Health betweenness centrality [>0.268] | -0.051 | 0.738 | 0.945 | -1.497 | 1.396 | 0.249 | 1.008 | 0.805 | -1.729 | 2.227 | 0.186 | 0.946 | 0.844 | -1.670 | 2.041 |
| Close friends with CMD | 0.006 | 0.018 | 0.757 | -0.030 | 0.042 | -0.043 | 0.025 | 0.088 | -0.092 | 0.006 | -0.033 | 0.022 | 0.146 | -0.077 | 0.011 |
| Trusts CMD for health advice | 0.017 | 0.015 | 0.253 | -0.012 | 0.047 | 0.043 | 0.020 | 0.034 | 0.003 | 0.082 | 0.040 | 0.018 | 0.028 | 0.004 | 0.075 |
| Age in years | 0.006 | 0.001 | <0.001 | 0.005 | 0.007 | 0.004 | <0.001 | <0.001 | 0.003 | 0.004 | 0.005 | <0.001 | <0.001 | 0.004 | 0.006 |
| Female | -0.005 | 0.010 | 0.583 | -0.025 | 0.014 | 0.019 | 0.012 | 0.103 | -0.004 | 0.041 | 0.014 | 0.011 | 0.206 | -0.007 | 0.034 |
| Social status: was or is a religious, tribe, or clan leader or on the local village council | -0.053 | 0.033 | 0.110 | -0.119 | 0.012 | -0.075 | 0.034 | 0.025 | -0.141 | -0.010 | -0.077 | 0.030 | 0.011 | -0.136 | -0.017 |
| Fisherman or fishmonger | -0.031 | 0.025 | 0.222 | -0.080 | 0.019 | 0.010 | 0.027 | 0.695 | -0.042 | 0.063 | -0.001 | 0.025 | 0.972 | -0.049 | 0.047 |
| Other income-earning occupation | -0.068 | 0.018 | <0.001 | -0.103 | -0.034 | -0.078 | 0.018 | <0.001 | -0.114 | -0.042 | -0.084 | 0.017 | <0.001 | -0.117 | -0.051 |
| Highest level of education attained | 0.030 | 0.002 | <0.001 | 0.026 | 0.034 | 0.024 | 0.002 | <0.001 | 0.020 | 0.029 | 0.029 | 0.002 | <0.001 | 0.025 | 0.033 |
| Muslim household head | 0.013 | 0.017 | 0.418 | -0.019 | 0.046 | 0.028 | 0.022 | 0.203 | -0.015 | 0.072 | 0.027 | 0.020 | 0.181 | -0.013 | 0.067 |
| Household head belongs to village majority tribe | 0.017 | 0.015 | 0.256 | -0.013 | 0.047 | 0.035 | 0.020 | 0.076 | -0.004 | 0.074 | 0.034 | 0.018 | 0.058 | -0.001 | 0.069 |
| No home latrine | 0.012 | 0.027 | 0.642 | -0.040 | 0.065 | 0.031 | 0.036 | 0.387 | -0.040 | 0.103 | 0.029 | 0.033 | 0.377 | -0.036 | 0.094 |
| Home owned, not rented | 0.056 | 0.023 | 0.017 | 0.010 | 0.101 | 0.058 | 0.030 | 0.051 | <-0.001 | 0.116 | 0.064 | 0.027 | 0.017 | 0.011 | 0.116 |
| Home quality score | 0.004 | 0.002 | 0.087 | -0.001 | 0.008 | 0.006 | 0.003 | 0.042 | <0.001 | 0.012 | 0.006 | 0.003 | 0.024 | 0.001 | 0.011 |
| Village centre greater than 0.50 km from Lake Victoria | -0.019 | 0.018 | 0.298 | -0.054 | 0.017 | -0.047 | 0.022 | 0.032 | -0.091 | -0.004 | -0.044 | 0.020 | 0.029 | -0.083 | -0.005 |
| Total homes in village | <0.001 | <0.001 | 0.526 | <-0.001 | <0.001 | <-0.001 | <0.001 | 0.005 | -0.001 | <-0.001 | <-0.001 | <0.001 | 0.019 | <-0.001 | <-0.001 |
| Obs. 7254^b^ |  |  |  |  |  |  |  |  |  |  |  |  |  |  |  |
| Households 2159^b^ |  |  |  |  |  |  |  |  |  |  |  |  |  |  |  |
| Inverse hyperbolic tangent of 𝜌 | 0.729 | 0.040 | <0.001 | 0.651 | 0.807 |  |  |  |  |  |  |  |  |  |  |
| 𝜌 | 0.622 | 0.024 |  | 0.572 | 0.668 |  |  |  |  |  |  |  |  |  |  |
| Wald test of independent equations, Chi^2^ | 328.75 |  | <0.001 |  |  |  |  |  |  |  |  |  |  |  |  |

^a^Entered in this bivariate probit model as grand-mean centered and a quadratic, i.e. also including the squared form. All covariate functional forms were determined using locally weighted regression.

^b^ Noncompliers were treated as missing. Only individuals offered at least one drug were included.

## Table 12: Two-sample t-tests for centrality against formal social status

|  |  | ***Without social status*** | | | ***With social status*** | | | | | | |
| --- | --- | --- | --- | --- | --- | --- | --- | --- | --- | --- | --- |
|  | **Variable** | **Obs.** | **Mean** | **Std. Err.** | **Obs.** | **Mean** | **Std. Err.** | **t** | **p-value** | **df** |  |
| **Individual level** | LN(Friendship degree +1) | 14008 | 2.252 | 0.005 | 423 | 2.458 | 0.035 | -6.423 | <0.001 | 14429 |  |
|  | Friendship closeness | 14008 | 0.382 | 0.001 | 423 | 0.408 | 0.004 | -7.394 | <0.001 | 14429 |  |
| **Household level** | LN(Friendship degree +1) | 2817 | 2.066 | 0.012 | 265 | 2.477 | 0.044 | -9.881 | <0.001 | 3080 |  |
|  | Friendship closeness | 2817 | 0.367 | 0.001 | 265 | 0.410 | 0.005 | -9.425 | <0.001 | 3080 |  |

## Table 13: Relationship with most trusted person for health advice

| **Relationship type** | **Freq.** | **Percent** |
| --- | --- | --- |
| Geographical neighbour | 704 | 24.45 |
| Close friend who I see frequently | 550 | 19.10 |
| Friend who I see sometimes | 57 | 1.98 |
| Local council member | 404 | 14.03 |
| Village health team member^a^ | 799 | 27.75 |
| Family that does not live in my household | 246 | 8.54 |
| Co-worker | 47 | 1.63 |
| Someone I go to church or mosque with | 36 | 1.25 |
| Stranger | 36 | 1.25 |
| **Total** | 2879 | 100 |

^a^ Includes community medicine distributors and other (3 per village) health team members.

In the health advice networks, individuals were asked to name people in the order of who they trusted the most and who they would first approach. Here, the types of relationships of the first named person, i.e. who the respondent would first turn to for advice about health problems, is presented. Self-loops, which occurred if someone named someone within their own home, were excluded. Respondents were allowed to specify multiple relationships with the named person. The relationship type was available for 79.71% (2616/3282) of households that provided at least one nomination in the health advice networks.
